# EHMT2 aggravates vascular remodeling via epigenetic inhibition of GADD45G

**DOI:** 10.1038/s12276-026-01702-6

**Published:** 2026-05-01

**Authors:** Zelan Wang, Junyong Zhao, Wenjian Luo, Ning Sun, Xingyu Ma, Fangyuan Zhong, Boji Wu, Heng Tang, Ke Ning, Jingyu He, Xuhong Wang, Kun Zhang, Jihang Zhang, Chuan Liu, Jun Ren, Yan Zhao, Zhexue Qin

**Affiliations:** 1 Department of Cardiology, Xinqiao Hospital, Army Medical University (Third Military Medical University), Chongqing, China; 2 Department of Cardiology, University-town Hospital of Chongqing Medical University, Chongqing, China; 3 Department of Pathogenic Biology, College of Basic Medical Sciences, Army Medical University (Third Military Medical University), Chongqing, China; 4 Department of Cardiology, Zhongshan Hospital, Fudan University, Shanghai Institute of Cardiovascular Diseases, Shanghai, China; 5 State Key Laboratory of Cardiovascular Diseases, Zhongshan Hospital, Fudan University, Shanghai, China; 6 Department of Microbiology, College of Basic Medical Sciences, Key Laboratory of Microbial Engineering Under the Educational Committee in Chongqing, Army Medical University (Third Military Medical University), Chongqing, China; 7 Basic Research Innovation Center for Acute Radiation Syndrome, Ministry of Education of the People’s Republic of China, Chongqing, China

**Keywords:** Valvular disease, Myocardial infarction

## Abstract

Euchromatic histone-lysine *N*-methyltransferase 2 (EHMT2) has been implicated in cardiovascular diseases, yet its role in vascular remodeling remains incompletely understood. Here we investigated the contribution of EHMT2 to vascular smooth muscle cell (VSMC) proliferation, migration and neointima formation following vascular injury using carotid artery injury models and in vitro VSMC studies. Transcriptomic (RNA sequencing) and epigenomic (CUT&Tag) profiling revealed that EHMT2 levels were elevated in injured arteries and growth-stimulated VSMCs, whereas EHMT2 deletion attenuated injury-induced neointima formation. Mechanistically, EHMT2 methyltransferase activity promoted VSMC proliferation and migration, with pathway analyses implicating cell cycle and growth programs as major downstream targets. We further identified GADD45G as a critical EHMT2-regulated gene characterized by H3K9me2 enrichment, and demonstrated that GADD45G enforced G1-phase arrest by suppressing cyclinB1, cyclinD1, CDK2 and CDK4. Importantly, both genetic and pharmacological inhibition of EHMT2, through GADD45G knockdown or administration of the EHMT2 inhibitor BIX-01294, significantly reduced neointimal lesion formation in injury models. These findings collectively establish EHMT2 as a key epigenetic driver of vascular remodeling by repressing GADD45G and facilitating cell cycle progression, highlighting EHMT2 as a potential therapeutic target for vascular proliferative diseases.

## Targeting EHMT2 reduces neointimal lesion formation

Neointima formation, a key feature of vascular remodeling, involves the proliferation and migration of vascular smooth muscle cells (VSMCs). This study investigates the role of the histone methyltransferase EHMT2 in regulating VSMC behavior during neointima formation. Using mouse models with smooth muscle cell-specific EHMT2 deletion, the authors demonstrate that EHMT2 deficiency significantly reduces neointima formation following vascular injury. The study uses transcriptome and CUT&Tag profiling to reveal that EHMT2 modulates VSMC proliferation by catalyzing H3K9 dimethylation at the GADD45G promoter, preventing cell cycle arrest. Results show that EHMT2 upregulation enhances VSMC proliferation and migration, while its inhibition reduces these processes, highlighting the methyltransferase activity’s importance. These findings suggest that targeting the EHMT2–GADD45G axis could offer new therapeutic strategies for vascular diseases characterized by abnormal VSMC proliferation and migration.

This summary was initially drafted using artificial intelligence, then revised and fact-checked by the author.

## Translational perspective

In this study, we demonstrate that EHMT2 plays a critical role in regulating VSMC proliferation and migration and in promoting injury-induced vascular neointima formation. Mechanistically, EHMT2 drives the pathological proliferation and migration of VSMCs through an epigenetic mechanism, namely lysine 9 on histone 3 (H3K9) dimethylation at the GADD45G locus. Collectively, these findings identify the EHMT2–GADD45G axis as a potential therapeutic target for preventing or treating pathological vascular remodeling.

## Introduction

Neointima formation, also referred to as intimal hyperplasia, is a common feature of vascular remodeling, including postangioplasty stenosis^1^, atherosclerosis^2^ and vein bypass graft failure^3,4^. Compelling evidence has demonstrated that, in vascular remodeling, the vascular smooth muscle cell (VSMC) phenotype switches from a contractile phenotype to a synthetic phenotype while exhibiting decreased expression of contractile proteins and increased proliferation and migration abilities^5^. Therefore, an improved understanding of the mechanisms underlying VSMC proliferation and migration may be crucial for the development of novel therapeutics for vascular pathologies.

Accumulating evidence highlights the importance of epigenetic modifications in the pathological processes of VSMC proliferation and neointima formation^6^. Histone methylation is an important determinant of chromatin spatial structure, and the regulation of chromatin conformation and transcription factor accessibility to DNA is crucial in VSMC fate switching^7,8^. However, the nature of histone methylation modification factors and how such factors favor VSMC proliferation during neointima formation are still poorly understood.

The histone methyltransferase euchromatic histone-lysine *N*-methyltransferase 2 (EHMT2), also known as G9a, is a su(var)3-9/enhancer of zeste/trithorax (SET) domain-containing protein with histone lysine methyltransferase activity^9^. EHMT2 is responsible for the monomethylation and dimethylation of lysine 9 on histone H3 (H3K9me1 and H3K9me2), resulting in transcriptional gene activation or repression^10^. EHMT2 is involved in cell development, pluripotency, cell proliferation and migration^11^. Aberrant EHMT2 expression is frequently observed in multiple tumors and contributes to cancer pathogenesis and progression^12^. Recently, EHMT2 was shown to play a critical role in cardiovascular remodeling. For example, EHMT2 expression was increased in advanced human carotid atherosclerotic plaques, and EHMT2 inhibitors blocked the proliferation and migration of arterial smooth muscle cells (SMCs)^13^. EHMT2 and its partner protein GLP have been shown to ameliorate pulmonary vascular remodeling^14^. In addition, EHMT2 inhibits aortic SMC death by suppressing autophagy activation^15^. However, the role of EHMT2 in VSMC proliferation in vascular neointima formation remains unknown.

In this study, we explored the role of EHMT2 in the proliferation and migration regulation of VSMCs and vascular intimal hyperplasia. SMC-specific EHMT2 deletion in mice inhibited injury-induced neointima formation in wire-injured carotid arteries. In vitro and in vivo studies revealed that EHMT2 modulated VSMC proliferation and migration. Transcriptome and cleavage under targets and tagmentation (CUT&Tag) profiling revealed that EHMT2 exerted its effects by directly catalyzing the H3K9 dimethylation of GADD45G and eliminating the GADD45G-induced VSMC cell cycle arrest. Our findings provide evidence that EHMT2 plays a crucial role in the epigenetic regulation of GADD45G during neointima formation in response to vascular injury.

## Materials and methods

Expanded methods are available in the Supplementary Information.

### Mouse carotid artery wire injury model

The carotid arteries of these mice were subjected to wire injury as previously described^16,17^. In brief, 10–12-week-old male mice weighing 25–30 g were anesthetized by inhalation of 2% isoflurane. A 0.38-mm guidewire (C-SF-15-15; Cook) was inserted through the external carotid artery incision into the arterial lumen toward the aortic arch and pulled back and forth five times with a rotating motion. The sham littermate control mice underwent the same procedures without the incision and passage of the wire. The mice were intraperitoneally injected with BIX-01294 at an established dose of 5 mg/kg (once a week) for 28 days^18,19^. Dimethyl-sulfoxide-treated mice served as the control mice. Adeno-associated virus 9 (AAV) for SMC-specific transfection with a mouse *GADD45G* small interfering RNA (siRNA) (AAV-si-*GADD45G*) or negative control (AAV-si*Scrambled*) was generated by Genechem Biotech. AAV-si-*GADD45G* titers (5.0 × 10^12 ^vg/ml) or negative control virus was injected into the tail veins of male *EHMT2*^SMCKO^ mice^20^.

### Rat carotid artery injury model

Rat carotid artery neointima formation was induced by balloon angioplasty to the left common carotid artery as previously described^21^. The procedures were performed under sodium pentobarbital (50 mg/kg) anesthesia. To denudate the endothelium, a 2-Fr Fogarty balloon catheter (Cordis) was inserted into the common carotid, advanced to the aortic arch, inflated to produce moderate resistance and gradually withdrawn three times. The sham-operated rat carotid arteries served as the control. The lentivirus transfection into the injured vessels was performed as previously described^22^. In brief, for in vivo *EHMT2*-knockdown studies, 100 μl of sh-*EHMT2* lentivirus (1 × 10^8^ TU/ml, transducing units per ml) and the GFP lentivirus (1 × 10^8^ TU/ml) (GeneChem, Shanghai, China) were luminally delivered into the injured carotid artery and incubated for 30 min to enhance transfection. After the lentivirus solution removal, the external carotid artery was ligated to restore the blood flow. The arteries were collected at the indicated time points.

### Cell culture

VSMCs were isolated from the thoracic aorta of male Sprague–Dawley rats (150–180 g) using a collagenase I digestion method, as previously described^23^. The VSMCs were maintained in Dulbecco’s modified Eagle medium containing 10% fetal bovine serum, 100 U/ml penicillin and 100 μg/ml streptomycin. VSMCs at passages 3–8 and approximately 80% confluency were used for experiments.

### Vector construction and cell transfection

Lentivirus particles carrying either *EHMT2* cDNA, its variant p.Ala1130Ser, si-*EHMT2*, *GADD45G* cDNA and si-*GADD45G* were generated commercially (GeneChem). In brief, for construction of the lentiviral vector carrying the EHMT2 p.Ala1130Ser variant, the p.Ala1130Ser mutation was introduced using the QuikChange II XL Site-Directed Mutagenesis Kit (Agilent Tech).

For in vitro transfection, the recombinant lentivirus was directly transfected into VSMCs 72 h before the experiment. For the siRNA transfection, VSMCs cultured in six-well plates were transfected with 20 nM siRNA per well using Lipofectamine RNAiMAX reagent. The siRNAs targeting rat *GADD45G*, *EHMT2* and scrambled sequence were synthesized by Sangon Biotech.

### RNA-seq analysis

Total RNA was extracted from four independent samples of rat VSMCs subjected to indicated treatment. RNA sequencing (RNA-seq) analysis was performed at Majorbio Bio-Pharm Tech, with differential gene expression analysis performed using DESeq2 (adjusted *P* value <0.05 and |fold change| >1.5). The functional enrichment analyses of Gene Ontology (GO) terms and Kyoto Encyclopedia of Genes and Genomes (KEGG) pathways was subsequently carried out.

### CUT&Tag assay

CUT&Tag experiments were performed using the Hyperactive In-Situ ChIP Library Prep Kit for Illumina (TD901-02, Vazyme), as previously described with modifications^24^. In brief, 100,000 cells were incubated with activated concanavalin A-coated magnetic beads. The bead-bound cells were permeabilized and successively incubated with H3K9me2 primary antibody (ab176882, Abcam) and secondary antibody. The beads were resuspended in pAG-Tn5-containing wash buffer. Tagmentation reaction was then performed. Extracted DNA fragments were used for polymerase chain reaction (PCR) amplification to prepare the DNA library. The CUT&Tag libraries were sequenced on an Illumina NovaSeq platform at Novogene.

### CUT&Tag data analysis

The CUT&Tag sequencing generated the PE150 sequencing data. FASTQ files were processed to assess the quality of the raw sequencing data. Clean reads were aligned to the rat genome (National Center for Biotechnology Information reference) using Bowtie2. The aligned reads were indexed and sorted with samtools. Duplicate reads in the input were removed using Picard, and peak calling was performed with MACS2, excluding blacklist regions of the rat genome. DeepTools was used to calculate scores for genomic regions of interest and to generate heatmaps. BigWig tracks were viewed in the Integrative Genomics Viewer (IGV) browser.

### Statistical analysis

All the experimental data are expressed as means ± standard errors of the mean. Statistical analysis was performed using GraphPad Prism (GraphPad, version 9.4.0). The Shapiro–Wilk normality test was performed before data comparison. Differences in means between two groups were analyzed via an unpaired two-tailed Student’s *t*-test or the Mann–Whitney *U* test as appropriate. Comparisons among more than two groups were performed using one-way analysis of variance (ANOVA) or the nonparametric Kruskal–Wallis test. A value of *P* < 0.05 was considered statistically significant.

## Results

### EHMT2 expression is upregulated in carotid artery neointima

**Fig. 1:**
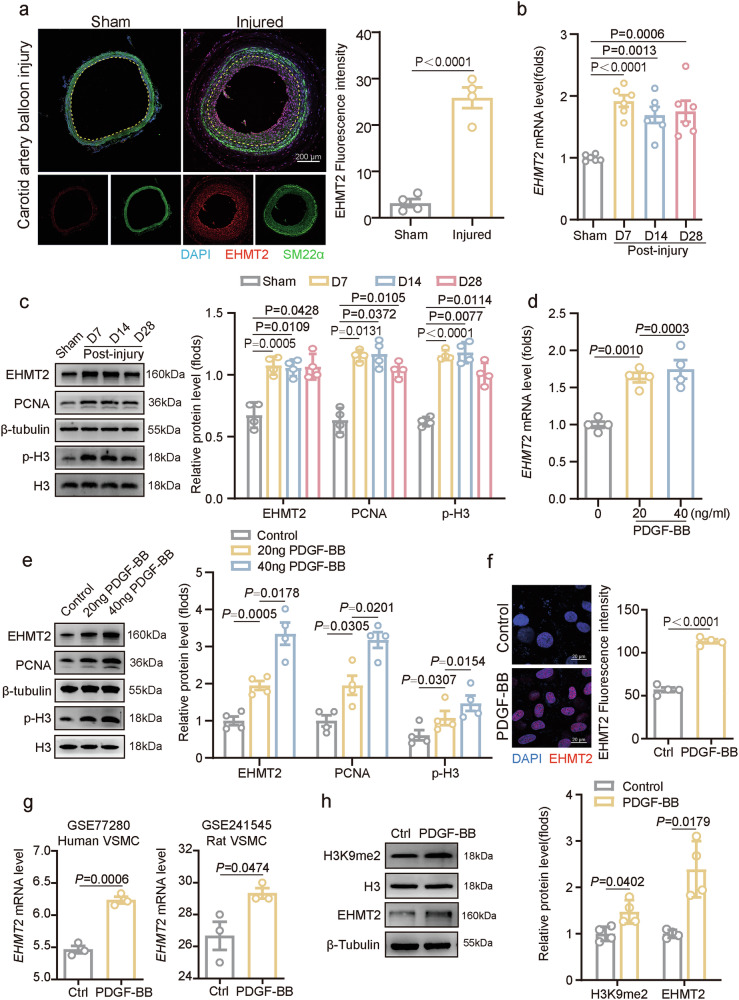
EHMT2 expression is upregulated in injured carotid arteries. **a** Immunofluorescence staining shows the expression of EHMT2 and SM22α in the carotid arteries (*n* = 4) at day 14 after balloon injury. Nuclei were stained with 4′,6-diamidino-2-phenylindole (DAPI). Scale bar, 200 µm. The intensity was normalized to that of DAPI. **b** Relative *EHMT2* mRNA levels in rat carotid arteries (*n* = 6) at different time points after balloon injury. The mRNA level was normalized to that of β-actin and quantified. **c** Relative protein levels of EHMT2, PCNA and p-H3 in balloon-induced carotid artery injury model rats (*n* = 4) at days 7, 14 and 28 postinjury were evaluated via western blot. **d** *EHMT2* mRNA expression was quantified in rat VSMCs (*n* = 4) stimulated with different concentrations of PDGF-BB for 24 h. **e** Relative protein levels of EHMT2, PCNA and p-H3 in rat VSMCs (*n* = 4) stimulated with PDGF-BB at concentrations of 20 ng/ml or 40 ng/ml were evaluated via western blot. **f** Immunofluorescence staining showed the localization and expression of EHMT2 in VSMCs in rat VSMCs (*n* = 4). Nuclei were stained with DAPI. Scale bar, 20 µm. The intensity was normalized to that of DAPI. **g** Quantitative analysis of *EHMT2* mRNA levels in rat VSMCs (*n* = 3) and human VSMCs (*n* = 3) obtained from the GEO database after PDGF-BB treatment. **h** Protein levels of H3K9me2 and EHMT2 in cultured VSMCs (*n* = 4) stimulated with PDGF-BB at a concentration of 20 ng/ml were evaluated via western blot. The data are presented as mean ± s.e.m. *P* values were calculated using an unpaired two-tailed *t*-test (**a** and **f**–**h**) or a one-way ANOVA (**b**–**e**).

To investigate the potential involvement of EHMT2 in the process of VSMC proliferation and neointima formation, we first analyzed EHMT2 expression in the rat balloon-injured carotid artery model. Cryosection staining of the carotid artery revealed that EHMT2 was mainly localized in the neointima and that EHMT2 expression was highly upregulated in the balloon-injured and wire-injured carotid neointima compared with the control at days 7 and 14 postinjury (Fig. 1a and Supplementary Fig. 1). Meanwhile, we found that the mRNA level of *EHMT2* was significantly increased on day 7 postoperation and was maintained at that level until day 28 (Fig. 1b). Similarly, the protein levels of EHMT2 and the proliferation markers PCNA and p-H3 were markedly increased in balloon-injured carotid arteries at days 7, 14 and 28 postinjury (Fig. 1c).

To recapitulate those findings in vitro, we analyzed *EHMT2* mRNA expression in cultured rat VSMCs in the presence of proliferative stimulus. Notably, platelet-derived growth factor-BB (PDGF-BB) is recognized as one of the most potent mitogens and chemokines acting on VSMCs and plays a central role in neointima formation after vascular injury^25,26^. Upon stimulation with different doses of PDGF-BB, *EHMT2* mRNA expression was upregulated in a dose-dependent manner (Fig. 1d). PDGF-BB promoted the upregulation of PCNA and p-H3, accompanied with elevated EHMT2 protein levels in a dose-dependent manner in cultured rat VSMCs (Fig. 1e). Immunofluorescence staining of cultured rat VSMCs revealed that EHMT2 was located in the nucleus and further condensed upon PDGF-BB stimulation (Fig. 1f). Furthermore, our observation was supported in Gene Expression Omnibus datasets. *EHMT2* mRNA expression was upregulated in PDGF-BB-stimulated human VSMCs in the GSE77280 dataset and in rat VSMCs in the GSE241545 dataset (Fig. 1g). In line with the H3K9me2 enzymatic activity of EHMT2, PDGF-BB treatment promoted the protein expression of both EHMT2 and H3K9me2 in VSMCs (Fig. 1h). Together, these results suggest that EHMT2 was aberrantly upregulated in synthetic VSMCs and neointima formation.

### SMC-specific EHMT2 deficiency attenuates neointima formation following vascular injury

**Fig. 2:**
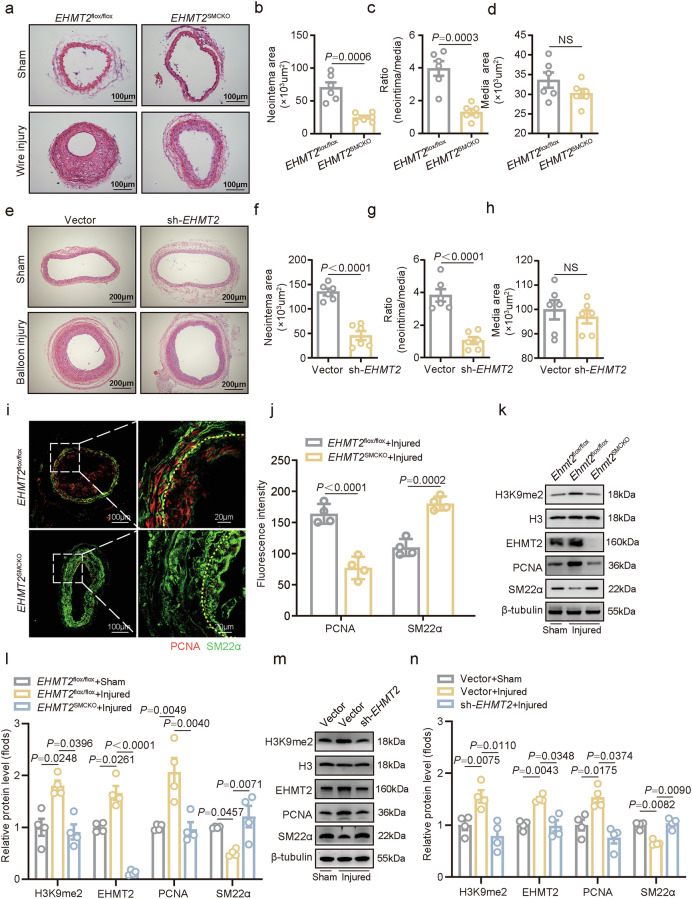
EHMT2 deficiency attenuates neointima formation following vascular injury. **a**–**d** Representative hematoxylin and eosin (HE) staining images (**a**) and quantification of the neointima area (**b**) intima-to-media ratio (**c**) and media area (**d**) on day 14 after carotid artery wire injury in *EHMT2*^flox/flox^ or *EHMT2*^SMCKO^ mice (*n* = 6). Scale bar, 100 µm. **e**–**h** Representative HE staining images (**e**) and quantification of the neointimal area (**f**) intima-to-media ratio (**g**) and media area (**h**) of the balloon-injured rat carotid artery (*n* = 6) with or without intraarterial transfection of lentivirus carrying vector or sh-*EHMT2* sequences at day 14 postinjury. Scale bar, 200 µm. **i**,**j** Immunofluorescence staining (**i**) and quantification of PCNA and SM22α in the carotid neointima (**j**) at day 14 after artery wire injury in *EHMT2*^flox/flox^ and *EHMT2*^SMCKO^ mice (*n* = 4). **k**,**l** The expression of EHMT2, H3K9me2, PCNA and SM22α was evaluated via western blot (**k**) and quantified (**l**) in the neointima of carotid artery after wire injury at day 14 postinjury in *EHMT2*^flox/flox^ or *EHMT2*^SMCKO^ mice (*n* = 4). **m**,**n** The expression of EHMT2, H3K9me2, PCNA and SM22α was evaluated via western blot (**m**) and quantified (**n**) in the neointima of carotid artery after balloon injury at day 14 postinjury in rats (*n* = 4) treated with lentivirus carrying vector or sh-*EHMT2* sequence. The data are presented as mean ± s.e.m. *P* values were calculated using an unpaired two-tailed *t*-test (**b**–**e**, **g**, **h** and **j**) or a one-way ANOVA (**l** and **n**).

To investigate whether EHMT2 contributes to neointima formation, SMC-specific *EHMT2*-knockout mice were constructed, followed by injury of the carotid artery with a wire (Supplementary Fig. 2). The SMC-specific deletion of *EHMT2* significantly decreased neointima formation in wire-injured carotid arteries in mice at day 14 postinjury (Fig. 2a), with a reduced area of neointima formation (Fig. 2b) and ratio of neointimal thickness to medial thickness (Fig. 2c) in the wire-injured carotid arteries compared with the control; however, the medial area was not notably altered (Fig. 2d).

In the rat balloon-injured carotid artery model, the local delivery of lentivirus carrying an *EHMT2* knockdown or empty vector was successfully performed. Compared with those in the empty vector group at day 14 postinjury, balloon injury-induced neointimal lesions were significantly reduced in the *EHMT2*-knockdown group after *EHMT2* virus transduction (Fig. 2e), as determined by decreases in both the neointimal area and the intima-to-media ratio (Fig. 2f,g). Moreover, the media area of the rat carotid artery was unaffected after *EHMT2* knockdown (Fig. 2h).

Meanwhile, immunofluorescence staining of PCNA and SM22α revealed that absence of EHMT2 decreased PCNA expression while increasing SM22α in carotid neointima (Fig. 2i,j). Western blot analysis confirmed that SMC-specific deletion of EHMT2 decreased the expression of H3K9me2 and PCNA but increased the expression of the VSMC contractile marker SM22α in mouse carotid arteries at day 14 (Fig. 2k,l). Consistently, the local delivery of EHMT2-knockdown lentivirus resulted in suppressed H3K9me2 and PCNA expression at day 14 in rat balloon-injured arteries (Fig. 2m,n). Taken together, these results suggest that EHMT2 acts as an important regulator of neointima formation.

### EHMT2 regulates the proliferation and migration of VSMCs

**Fig. 3:**
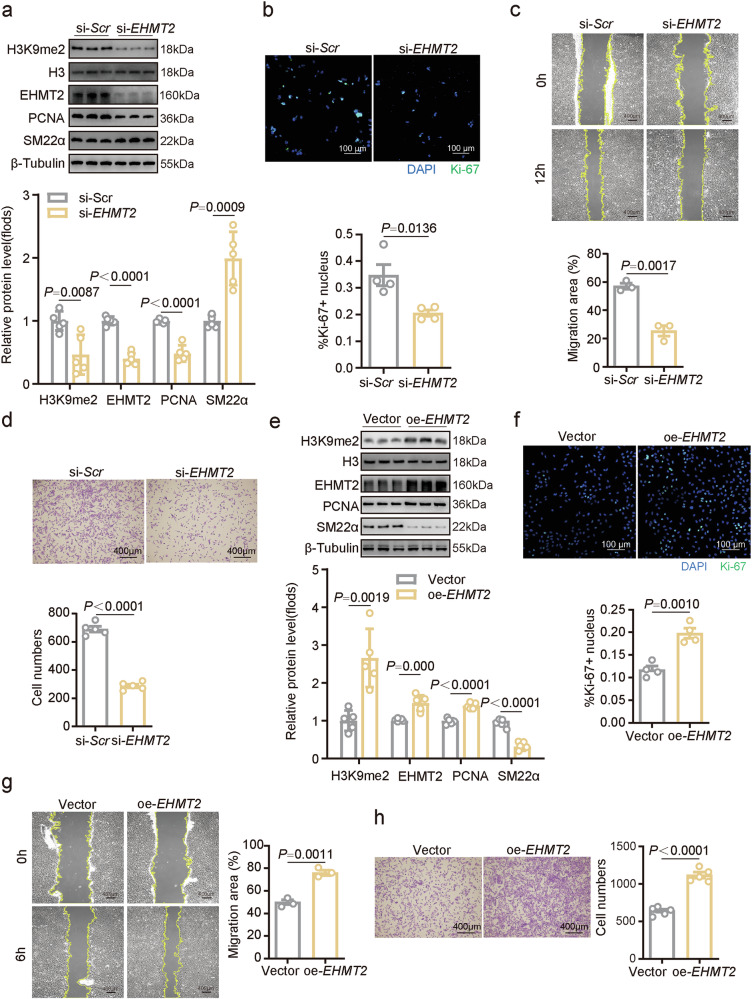
EHMT2 regulates the proliferation and migration of VSMCs in vitro. **a** Representative western blot analysis and quantification of EHMT2, H3K9me2, PCNA and SM22α expression in VSMCs transfected with si-*EHMT2* or si-*Scr* (*n* = 5). **b** VSMC proliferation (*n* = 4) was assessed via Ki-67 immunofluorescence. Nuclei were stained with DAPI. Scale bar, 100 μm. **c** Representative images of wound-healing assays. Images of si-*EHMT2* or si-*Scr* control-treated VSMCs (*n* = 4) were taken at 0 and 12 h after scratching. Scale bar, 200 µm. **d** Representative images and quantitative analysis of the migration of VSMCs (*n* = 5) treated with si-*EHMT2* or si-*Scr* control. Scale bar, 400 µm. **e** Representative western blot analysis and quantification of EHMT2, H3K9me2, PCNA and SM22α expression in VSMCs (*n* = 5) transfected with lentivirus carrying EHMT2 cDNA (oe-*EHMT2*) or control vectors. **f** VSMC proliferation (*n* = 4) was assessed via Ki-67 immunofluorescence, and the nuclei were stained with DAPI. Scale bar, 100 μm. **g** Representative images of wound-healing assays. Images of oe-*EHMT2* or vector control lentivirus-transfected VSMCs (*n* = 3) were taken at 0 and 6 h after scratching. Scale bar, 200 µm. **h** Transwell assays of VSMCs (*n* = 5) were performed after treatment with and without oe-*EHMT2*. Scale bar, 400 µm. The data are presented as mean ± s.e.m. *P* values were calculated using an unpaired two-tailed *t*-test (**a**–**h**).

To validate the abovementioned in vivo observations, we investigated whether EHMT2 affects VSMC proliferation in vitro. The siRNA effectively knocked down *EHMT2* expression in VSMCs (Supplementary Fig. 3), and *EHMT2* knockdown significantly suppressed the expression of H3K9me2 and PCNA, which was accompanied by the upregulation of SM22α expression (Fig. 3a). Furthermore, immunofluorescence assays for Ki-67, another well-recognized proliferation marker, revealed that *EHMT2* knockdown dramatically decreased the proliferation rate of VSMCs (Fig. 3b). Cell Counting Kit-8 (CCK-8) proliferation assay showed that *EHMT2* knockdown prevented PDGF-BB-evoked VSMC proliferation (Supplementary Fig. 4). Both scratch and transwell assays were performed to examine the role of EHMT2 in cell migration. Scratch assays revealed that the healing area was dramatically smaller in the *EHMT2*-knockdown group than in the control group upon PDGF-BB stimulation (Fig. 3c). Similarly, the transwell assay revealed fewer migratory cells in the *EHMT2*-knockdown group than the control group (Fig. 3d), suggesting that EHMT2 inhibition impairs cell migration ability.

The results of the above loss-of-function experiments prompted us to investigate whether *EHMT2* overexpression promoted the dedifferentiation of SMCs into a more proliferative state. Protein level analysis and fluorescence imaging revealed that EHMT2 was successfully overexpressed in VSMCs through lentiviral transduction (Supplementary Fig. 5). *EHMT2* overexpression increased levels of H3K9me2 and PCNA (Fig. 3e) and enhanced VSMC proliferation, as determined by Ki-67 immunofluorescence staining (Fig. 3f), while lowering the level of contractile marker SM22α (Fig. 3e). Compared with the control, the overexpression of *EHMT2* also promoted the migration of VSMCs, as determined by scratch (Fig. 3g) and transwell assays (Fig. 3h).

Moreover, two distinct EHMT2 chemical inhibitors, BIX-01294 and UNC0642, were used to determine the effects of EHMT2 inhibition. BIX-01294 and UNC0642 substantially suppressed EHMT2 enzymatic activity in cell-free systems^27^ (Supplementary Fig. 6). As expected, both BIX-01294 and UNC0642 effectively attenuated EHMT2 enzymatic activity in VSMCs (Supplementary Fig. 7a,b). BIX-01294 decreased VSMC proliferation as detected by CCK-8 analysis in a concentration-dependent manner in the presence of PDGF-BB (Supplementary Fig. 8). Furthermore, both BIX-01294 and UNC0642 inhibited the expression of VSMC proliferation markers PCNA and Ki-67, as well as VSMC migration in transwell assays, whereas EHMT2 overexpression partially reversed the inhibitory effects of these two inhibitors on proliferation and migration (Supplementary Fig. 7). Together, these observations suggest that EHMT2 mediates the proliferation and migration of VSMCs.

### EHMT2 promotes VSMC proliferation in a methyltransferase activity-dependent manner

**Fig. 4:**
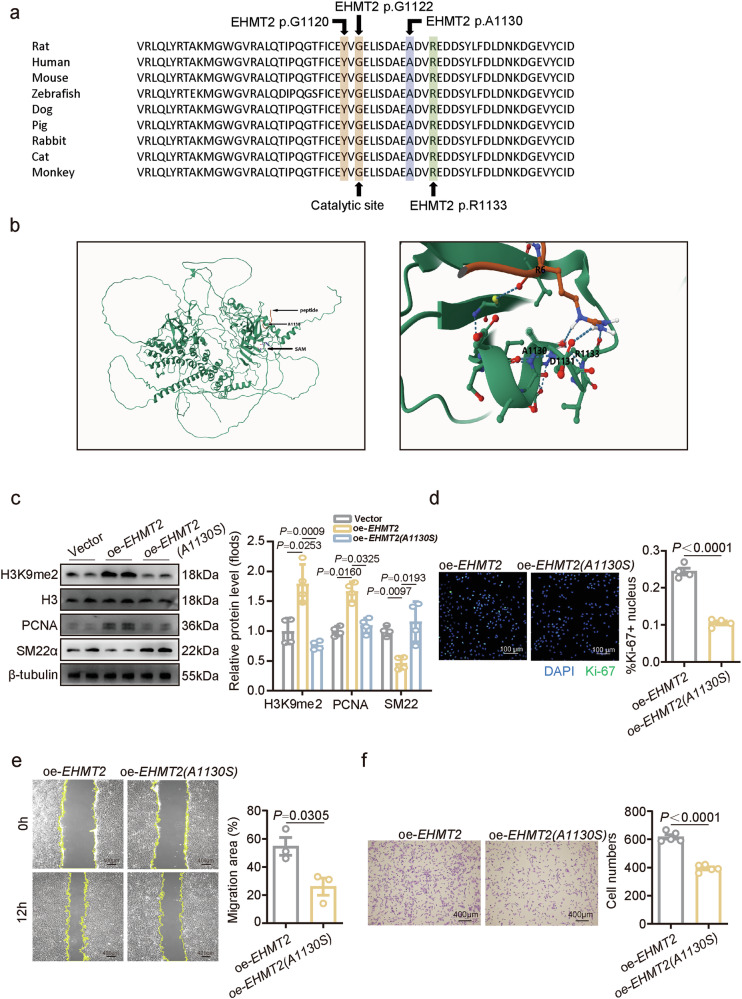
EHMT2 promotes VSMC proliferation in a methyltransferase activity-dependent manner. **a** Analysis of histone methyltransferase sequence alignment across different species, with a specific focus on the rat p.Ala1130 residue and other residues linked to enhanced functional activity. **b** Structure of EHMT2 SET domain dimers interacting with histone H3 and SAM. Magnification revealing that the formation of hydrogen bonds between residue p.Ala1130 in EHMT2 and the histone tail residue and other EHMT2 residues. **c** Representative western blot analysis and quantification of H3K9me2, SM22α and PCNA expression in oe-*EHMT2*, oe-*EHMT2*(A1130S) and control lentivirus vector-transfected VSMCs (*n* = 4). **d** VSMC (*n* = 4) proliferation was assessed via a Ki-67 assay, and nuclei were stained with DAPI. Scale bar, 100 μm. **e** Representative images of wound-healing assays. Images were taken at 0 and 12 h after scratching monolayers of oe-*EHMT2* VSMCs, oe-*EHMT2* (A1130S) VSMCs and control VSMCs (*n* = 3). **f** Representative images and quantitative analysis of the migration of VSMCs (*n* = 5) treated with oe-*EHMT2* and oe-*EHMT2* (A1130S). Scale bar, 400 µm. The data are presented as mean ± s.e.m. *P* values were calculated using an unpaired two-tailed *t*-test (**d**–**f**) or a two-way ANOVA (**c**).

EHMT2 is a histone methyltransferase that functions primarily through H3K9me2 in mammals^28^. To elucidate whether the role of EHMT2 in VSMCs is dependent on its methyltransferase activity, the key amino acid in the catalytic domain of EHMT2 was predicted and mutated. According to the published EHMT2 catalytic domain structure in complex with the H3 tail^29^, Ala 1130 of rat EHMT2, which is homogeneous to Ala1077 in humans, was predicted to be a mutation candidate to alter the catalytic activity of EHMT2 (Fig. 4a). Therefore, rat EHMT2 Ala 1130, which is located in the catalytic SET domain, was substituted with serine (p.Ala1130Ser; Fig. 4b). A lentivirus carrying the p.Ala1130Ser variant was transfected into cultured rat VSMCs. Compared with the EHMT2 group, the p.Ala1130Ser group presented significantly decreased total H3K9me2 levels (Fig. 4c). Moreover, p.Ala1130Ser overexpression led to a decrease in the VSMC proliferation marker PCNA and an increase in the contractile marker SM22α (Fig. 4c). Furthermore, the p.Ala1077Ser variant reduced the percentage of Ki-67-positive VSMCs (Fig. 4d). Similarly, the results of the scratch and transwell assays revealed that the p.Ala1130Ser variant inhibited the migratory ability of VSMCs (Fig. 4e,f). Overall, the regulatory effect of EHMT2 on VSMC proliferation and migration is dependent on its methyltransferase activity.

### Identification of GADD45G as an EHMT2-mediated H3K9me2-targeted protein in VSMCs

**Fig. 5:**
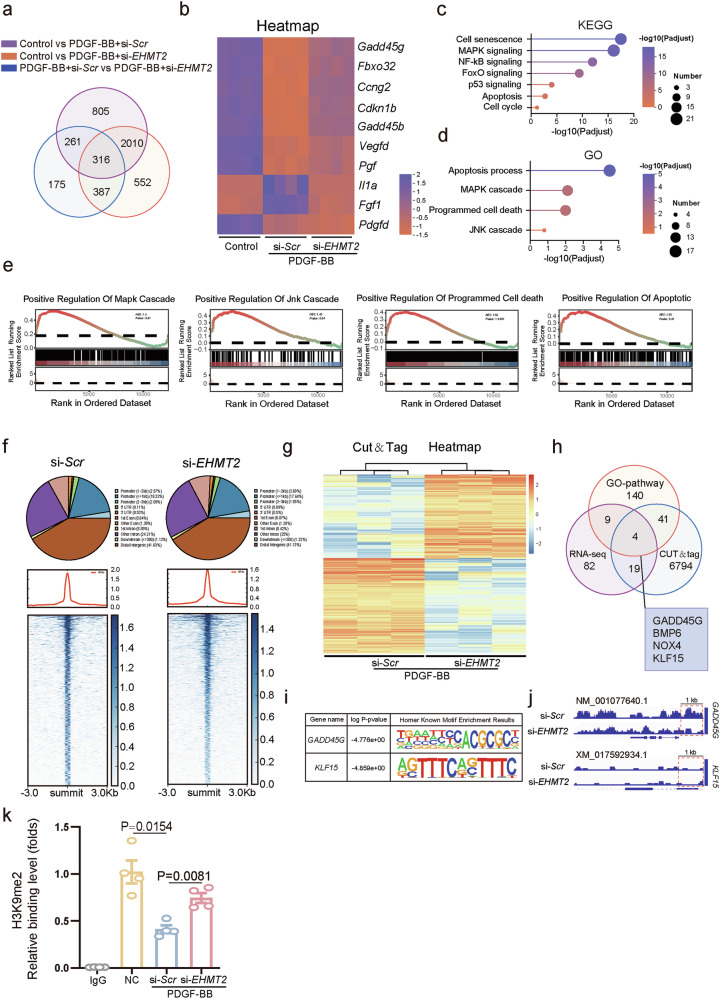
EHMT2 targets H3K9me2 to modulate the transcription and translation of GADD45G. **a** Venn diagram showed the number of DEGs uniquely identified in each comparison. **b** Heatmap of the transcriptomic profiling of the DEGs. **c**,**d** KEGG (**c**) and GO (**d**) analyses of DEGs in VSMCs. **e** GSEA of DEGs in VSMCs treated as indicated within pathways related to cell death and growth. **f** Pie chart displaying the genome-wide distribution of H3K9me2 occupancy in promoter, exon, intron and intergenic regions in VSMCs derived from CUT&Tag data. **g** Heatmap of the target genes associated with differential H3K9me2 peak binding sites in si-*EHMT2* and si-*Scr* VSMCs following PDGF-BB stimulation. **h** Venn diagram depicting the intersection of genes under EHMT2 regulation, genes bound by H3K9me2 and target genes identified via GO pathway analysis of the RNA-seq data. **i** Analysis of motif interactions with enriched H3K9me2 peaks at the GADD45G and Klf15 loci. **j** IGV analysis of H3K9me2 peaks at the GADD45G and Klf15 loci in VSMCs. **k** ChIP–qPCR assay was performed to detect the relative binding level of H3K9me2 at the promoter region of GADD45G in VSMCs under different treatments.

To elucidate the underlying mechanisms by which EHMT2 regulates VSMC proliferation, the transcriptome of VSMCs treated with PDGF-BB in the presence or absence of *EHMT2* siRNA was profiled via RNA-seq. Notably, principal component analysis revealed significant differences in gene expression patterns between the control and *EHMT2*-knockdown groups (Supplementary Fig. 9). PDGF-BB-treated VSMCs presented 1,382 differentially expressed genes (DEGs), whereas VSMCs cotreated with PDGF-BB and *EHMT2* siRNA presented 752 genes with altered expression levels, resulting in 316 genes reciprocally upregulated by PDGF-BB and downregulated by *EHMT2* siRNA (Fig. 5a). Genes ranked according to their association with proliferation or functional relevance to proliferation are displayed in a heatmap (Fig. 5b). KEGG and GO enrichment analyses revealed that multiple proliferation-related pathways, including cell cycle, cell growth, cell differentiation and apoptosis, were influenced by EHMT2 (Fig. 5c,d). Subsequent gene set enrichment analysis (GSEA) revealed that cell death and growth pathways, including MAPK cascade and JNK signaling, were enriched upon *EHMT2* knockdown (Fig. 5e). These data further support that EHMT2 facilitates the growth of VSMCs.

Given that EHMT2 regulates H3K9me2 in mammals, CUT&Tag analysis was performed to identify candidate genes downstream of EHMT2. Global mapping analysis revealed that *EHMT2* knockdown altered the H3K9me2 binding region distribution in the genome (Fig. 5f). In 3 replicates, 51,749 H3K9me2 interaction sites were identified after normalization with CUT&Tag. *EHMT2* knockdown decreased the H3K9me2 signals of VSMCs, as shown in the general CUT&Tag data heatmaps (Fig. 5g), with a decrease of 13,958 H3K9me2 peak binding sites.

To further identify potential target genes of H3K9me2, we intersected genes showing hypomethylation in CUT&Tag data with upregulated genes from RNA-seq analysis. This approach identified 23 DEGs with associated H3K9me2 binding peaks (Fig. 5h). GO analysis for proliferation-related functions among these 23 DEGs highlighted four candidate genes: GADD45G, Bmp6, Nox4 and Klf15 (Fig. 5h). Subsequent motif analysis of H3K9me2 CUT&Tag-seq peaks revealed enrichment for canonical motifs corresponding to GADD45G and Klf15 (Fig. 5i), and IGV analysis confirmed H3K9me2 binding at the promoter regions of these two genes (Fig. 5j). While Klf15 is known to maintain the contractile phenotype of VSMCs and inhibit neointima formation, bioinformatic analysis indicated that EHMT2 binds specifically to the GADD45G promoter (Fig. 5j). This binding was functionally relevant, as EHMT2 knockdown reduced H3K9me2 enrichment at the GADD45G promoter (Fig. 5j). Based on these findings, we performed chromatin immunoprecipitation followed by quantitative PCR (ChIP–qPCR), which confirmed that EHMT2 directly regulates GADD45G transcription via H3K9me2 (Fig. 5k and Supplementary Fig. 10). Collectively, these data indicate that GADD45G is a downstream target modulated by EHMT2-mediated H3K9me2 during vascular remodeling.

### GADD45G, which is negatively regulated by EHMT2, induces G1 cell cycle arrest during VSMC proliferation

**Fig. 6:**
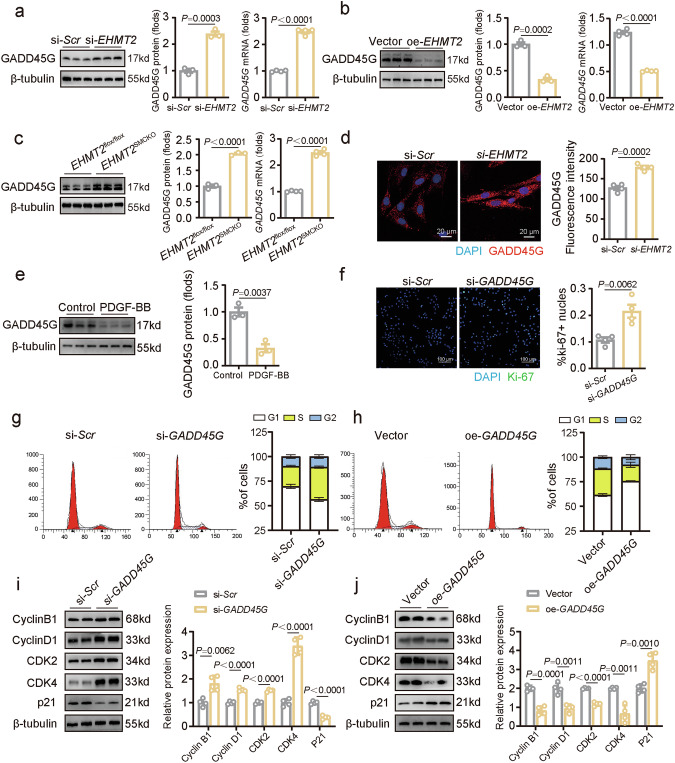
GADD45G regulates the G1/S phase transition in the VSMC cell cycle. **a** The mRNA and protein levels of GADD45G in rat VSMCs (*n* = 4) treated with si-*Scr* or si-*EHMT2*. **b** The mRNA and protein levels of GADD45G in rat VSMCs (*n* = 4) treated with lentivirus vector (Vector) or carrying EHMT2 (oe-*EHMT2*). **c** Representative western blot analysis and quantification of GADD45G expression in *EHMT2*^flox/flox^ and *EHMT2*^SMCKO^ mice (*n* = 3). **d** Immunofluorescence staining of GADD45G in VSMCs (*n* = 4) with si-*Scr* or si-*EHMT2*. Nuclei were stained with DAPI. Scale bar, 20 µm. The intensity was normalized to that of DAPI. **e** The expression of GADD45G in rat VSMCs (*n* = 4) stimulated with PDGF-BB or the control was evaluated via western blot. **f** VSMC proliferation (*n* = 4) was detected via Ki-67 immunofluorescence, and nuclei were stained with DAPI. Scale bar, 100 μm. **g** Percentages of VSMCs in different phases of the cell cycle after being treated or untreated with si-*EHMT2*. **h** Percentages of VSMCs in different phases of the cell cycle after being treated or untreated with oe-*EHMT2*. **i** Representative western blot analysis and quantification of cyclin B1, cyclin D1, CDK2, CDK4 and p21 expression in si-*GADD45G*-treated VSMCs and control VSMCs (*n* = 4). **j** Representative western blot analysis and quantification of cyclin B1, cyclin D1, CDK2, CDK4 and p21 expression in oe-*GADD45G* VSMCs and control VSMCs (*n* = 4). *P* values were calculated using an unpaired two-tailed *t*-test (**a**–**j**).

To validate the link between EHMT2 and GADD45G, we further investigated whether GADD45G expression was negatively associated with EHMT2 in VSMCs. Western blot and reverse transcription qPCR revealed that *EHMT2* knockdown increased GADD45G expression (Fig. 6a), whereas *EHMT2* overexpression downregulated GADD45G expression (Fig. 6b). In murine carotid arteries with SMC-specific EHMT2 deficiency, GADD45G expression was elevated (Fig. 6c). Immunofluorescence staining indicated that the GADD45G protein was mainly localized in the cytoplasm (Fig. 6d). Together with the CUT&Tag analysis results, we concluded that GADD45G is a target gene regulated by EHMT2-mediated H3K9 methylation in VSMCs.

Although GADD45G was shown to be involved in multiple GSEA pathways related to cell growth, it remains unknown whether GADD45G participates in the proliferation of VSMCs. GADD45G expression was downregulated upon PDGF-BB stimulation in VSMCs and in balloon injury-induced neointima as determined by western blot and immunostaining (Fig. 6e and Supplementary Fig. 11). *GADD45G* knockdown increased the number of Ki-67-positive VSMCs (Fig. 6f). Deregulation of the cell cycle in VSMCs is a universal hallmark of intimal hyperplasia and is considered a potential mechanism for inhibiting VSMC proliferation^30^. The cell cycle distribution of VSMCs was assessed via flow cytometry analysis, and we found that *GADD45G* knockdown increased the percentage of cells in the S phase (Fig. 6g). By contrast, the proportion of VSMCs in the S phase was significantly lower in the *GADD45G*-overexpressing group than in the vector control group (Fig. 6h). Moreover, the expression of the cell cycle markers cyclin B1, cyclin D1, CDK2, CDK4 and p21 was screened. *GADD45G* knockdown suppressed p21 expression and promoted cyclin B1, cyclin D1, CDK2 and CDK4 expression (Fig. 6i). *GADD45G* overexpression induced the expression of p21 and decreased the levels of cyclin B1, cyclin D1, CDK2 and CDK4, leading to cell cycle arrest in VSMCs (Fig. 6j). These data demonstrate that GADD45G, regulated by EHMT2, arrests the VSMC cell cycle at the G1 phase, preventing progression into the S phase.

### EHMT2 regulates VSMC proliferation and migration through the negative regulation of GADD45G

**Fig. 7:**
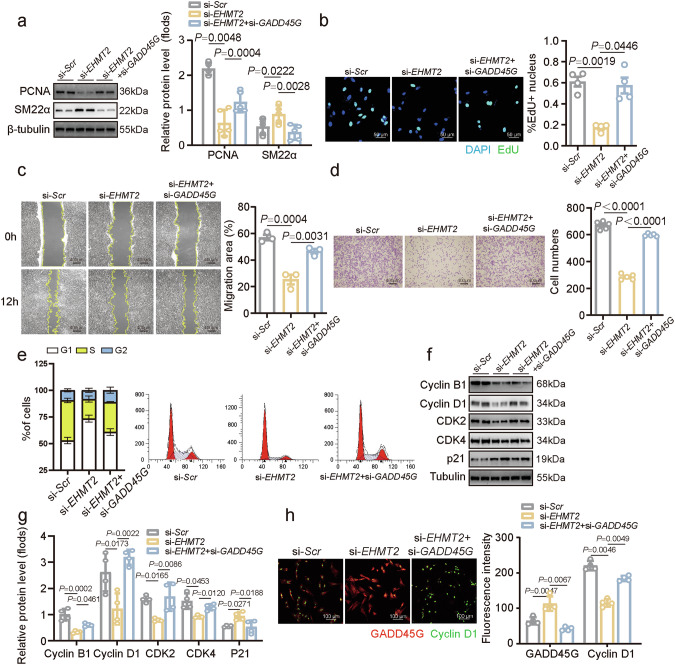
EHMT2 regulates VSMC proliferation and migration via the negative regulation of GADD45G. **a** Representative western blot analysis and quantification of PCNA and SM22α expression in VSMCs treated with si-*Scr*, si-*EHMT2* and combined EHMT2/GADD45G knockdown (si-*EHMT2* + si-*GADD45G*) (*n* = 5 in each group). **b** The proliferation of VSMCs (*n* = 4) was detected via immunostaining of EdU, and nuclei were stained with DAPI. Scale bar, 50 μm. **c** Representative images of wound-healing assays. Images were taken at 0 and 12 h after scratching monolayers in VSMCs treated with si-*Scr*, si-*EHMT2* and combined EHMT2/GADD45G knockdown (si-*EHMT2* + si-*GADD45G*) (*n* = 3 in each group). **d** Representative images and quantitative analysis of transwell migration assay in VSMCs treated with si-*Scr*, si-*EHMT2* and combined EHMT2/GADD45G knockdown (si-*EHMT2* + si-*GADD45G*) (*n* = 5 in each group). Scale bar, 400 µm. **e** Percentages of VSMCs in different phases of the cell cycle after treatment with si-*Scr*, si-*EHMT2* and combined EHMT2/GADD45G knockdown (si-*EHMT2* + si-*GADD45G*) (*n* = 5 in each group). **f**,**g** Representative western blot analysis (**f**) and quantification (**g**) of cyclin B1, cyclin D1, CDK2, CDK4 and p21 expression in VSMCs treated with si-*Scr*, si-*EHMT2* and combined EHMT2/GADD45G knockdown (si-*EHMT2* + si-*GADD45G*) (*n* = 4 in each group). **h** Immunofluorescence staining showed the expression of GADD45G and cyclin D1 in si-*Scr*, si-*EHMT2* and combined EHMT2/GADD45G knockdown (si-*EHMT2* + si-*GADD45G*) transfected VSMCs (*n* = 4 in each group). Nuclei were stained with DAPI. Scale bar, 100 µm. The intensity was normalized to that of DAPI. *P* values were calculated using a one-way ANOVA (**a**–**h**).

To determine whether GADD45G mediates the inhibition of proliferation induced by *EHMT2* silence, *GADD45G* was knocked down in VSMCs. *GADD45G* silencing abrogated the inhibitory effects of si-*EHMT2* on VSMC proliferation, as determined by PCNA expression, EdU incorporation and CCK-8 analysis (Fig. 7a,b and Supplementary Fig. 12). Similarly, scratch and transwell assays revealed that the simultaneous knockdown of *GADD45G* and EHMT2 restored the decreased migratory ability of VSMCs after *EHMT2* knockdown (Fig. 7c,d).

Given the regulation of cell cycle by GADD45G, we investigated whether EHMT2 and GADD45G are involved in the process of VSMC cell cycle arrest. Flow cytometry analysis of proportions of VSMCs revealed that *EHMT2* knockdown induced G1 cell cycle arrest in VSMCs, an effect that was alleviated by the transfection with *GADD45G* siRNA (Fig. 7e). The screening of cell cycle markers revealed that *EHMT2* knockdown led to the upregulation of p21 expression and the downregulation of cyclin B1, cyclin D1, CDK2 and CDK4 expression in VSMCs (Fig. 7f,g). Concomitant knockdown of *GADD45G* partially reversed these changes induced by *EHMT2* silencing alone (Fig. 7f,g). Immunostaining of cyclin D1 suggested that *GADD45G* knockdown reversed the suppressed expression of cyclin D1 after *EHMT2* knockdown (Fig. 7h). Taken together, these results suggest that the epigenetic suppression of *GADD45G* is an important downstream mechanism for EHMT2-mediated VSMC proliferation and migration.

### Potential therapeutic effects of targeting the EHMT2–GADD45G axis in vascular remodeling

**Fig. 8:**
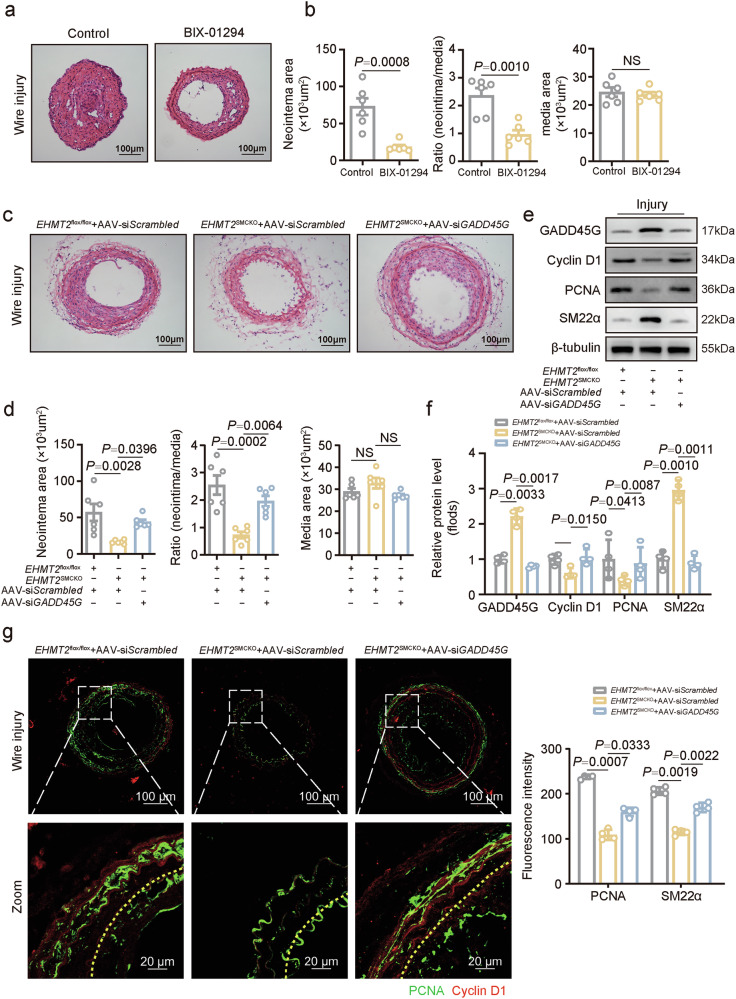
Potential therapeutic effects of targeting the EHMT2–GADD45G axis in vascular remodeling. **a**,**b** Representative images of HE-stained tissues (**a**) and quantification of neointima area and the intima-to-media ratio (**b**) on day 28 after wire injury of the carotid arteries of mice (*n* = 6) treated with or without BIX-01294. Scale bar, 100 µm. **c**,**d** Representative images of HE-stained tissues (**c**) and quantification of neointima area and the intima-to-media ratio (**d**) on day 14 after wire injury of the carotid arteries of the *EHMT2*^flox/flox^ or *EHMT2*^SMCKO^ mice (*n* = 6) transfected with AAV-si-scrambled or AAV-si-*GADD45G*. Scale bar, 100 µm. **e**,**f** Expression of GADD45G, cyclin D1, PCNA and SM22α in the carotid neointima (*n* = 3) after wire injury was evaluated via western blot (*n* = 3). **g** Immunofluorescence staining and quantification of PCNA and cyclin D1 in the carotid neointima (*n* = 4) at day 14 after wire injury. Scale bar, 20 µm. All data are presented as mean ± s.e.m. *P* values were calculated using an unpaired two-tailed *t*-test (**b**) or a one-way ANOVA (**d**, **f** and **g**).

To test the potential therapeutic value of EHMT2 for vascular remodeling, EHMT2 was pharmacologically inhibited by BIX-01294 to assess changes in intimal hyperplasia. Consistent with the results of the genetic insufficiency experiments, BIX-01294 treatment, compared with control treatment, led to a substantial decrease in the neointima area and the intima-to-media ratio at 28 days postinjury (Fig. 8a,b). Meanwhile, protein levels of H3K9me2 and PCNA were downregulated in murine carotid arteries following BIX-01294 treatment (Supplementary Fig. 13).

Moreover, to validate the involvement of GADD45G in neointima formation after carotid artery injury, we intravenously injected adeno-associated viruses targeting VSMCs carrying siRNA against *GADD45G* into tail veins of *EHMT2*-knockout mice. We found that neointima formation was significantly restored by *GADD45G* knockdown at day 14 postinjury in *EHMT2*-knockout mice (Fig. 8c,d), together with increased protein levels of PCNA and cyclin D1, as well as decreased protein levels of GADD45G and SM22α, in injured carotid arteries (Fig. 8e,f). In addition, the inhibition of VSMC proliferation after *EHMT2* knockout was alleviated after *GADD45G* knockdown, as shown by immunofluorescence staining for PCNA and cyclin D1 (Fig. 8g), suggesting that *GADD45G* overexpression might be a potential therapeutic strategy for neointima formation. Collectively, these findings indicate that the EHMT2–GADD45G axis represents a promising avenue for the development of novel preventive and therapeutic strategies for the treatment of vascular remodeling.

## Discussion

Preventing restenosis following vascular injury is crucial for enhancing the therapeutic efficacy, prognosis and quality of life of patients with coronary artery disease after stent implantation. Here, we demonstrated that the targeted modulation of EHMT2 significantly reduces neointima formation induced by carotid artery injury. In this study, we confirmed that EHMT2 expression was upregulated in PDGF-BB-stimulated VSMCs and injured carotid arteries. In vivo, the targeted depletion of VSMC EHMT2 resulted in a reduction in injury-induced neointima formation in the carotid artery. Our findings revealed that the inhibition of EHMT2 led to a decrease in the proliferation and migration functions of VSMCs and a reduction in the proportion of cells in S phase; moreover, these biological functions were strongly dependent on the methyltransferase activity of EHMT2. Our data also demonstrated that EHMT2 modulates VSMC growth partially through histone methylation-mediated regulation of GADD45G and its involvement in cell cycle-related biological processes. In addition, the inhibition of GADD45G rescued VSMC proliferation and neointima formation ability after carotid artery injury in the context of EHMT2 depletion or deficiency. In conclusion, EHMT2 may represent a novel therapeutic target for vascular diseases associated with the abnormal proliferation and migration of VSMCs.

The expression of H3K9me2 has been reported to be lower in atherosclerotic plaques from patients compared with healthy human arteries^13^. Furthermore, Liang et al. profiled histone methylation via quantitative mass spectrometry and revealed that H3K9me2 expression was downregulated in cultured human VSMCs upon PDGF-BB stimulation^31^. Specifically, H3K9me2 was found to be reduced within VSMCs in atherosclerosis and injury-induced arterial remodeling via the use of in vivo VSMC lineage tracing systems^32^. Similarly, H3K9me2 levels were lower in VSMCs from patients with diabetes than in those from control patients^33,34^. These observations consistently illustrate the loss of H3K9me2 in VSMCs during atherosclerosis and vascular neointimal hyperplasia. Moreover, H3K9me2 is enriched in silenced genes and is related to decreased chromatin accessibility and transcriptional repression^28^. A reduction in H3K9me2 denotes activation of a series of genes favoring a more proliferative state in VSMCs. H3K9me2 is catalyzed mainly by EHMT2 rather than by EHMT1 (GLP) in mammals, and EHMT2 expression is upregulated during vascular remodeling. Data from the GEO database and the observations in our study, including those from VSMCs under PDGF stimulation and after balloon-induced carotid artery injury, were consistent with a previous report on the upregulation of EHMT2 expression in VSMCs from human carotid atherosclerotic lesions^12^. The apparent contradiction between decreased H3K9me2 and increased EHMT2 might be attributed to the fine and complicated balance between H3K9 methylation and demethylation by groups of histone lysine methylation modifiers. In addition, EHMT1 (GLP) and EHMT2 are structurally homologous and can form a functional heterodimer that catalyzes H3K9me2^35^. EHMT1 might therefore partially compensate for H3K9me2 deposition when EHMT2 is deleted or downregulated. Genetic or pharmacological strategies targeting both EHMT1 and EHMT2 have been developed to achieve more complete suppression of H3K9me2^36^. Accordingly, the use of BIX-01294 and UNC0642 in this study cannot fully rule out their potential inhibitory effects on EHMT1. Given the dynamic balance between methylation and demethylation and the functional redundancy between EHMT1 and EHMT2, we focused on EHMT2 to clarify its specific contribution to vascular remodeling.

Epigenetic regulation has been reported to be crucial during VSMC differentiation and neointima formation^37,38^. DNA methylation and DNA methyltransferases play pivotal roles in regulating vascular neointima formation and SMC plasticity^39^. Histone modifications, particularly acetylation and methylation, have been shown to be vital elements in the determination of VSMC plasticity. A growing body of evidence has shown that HDACs, histone acetyltransferases and methyltransferases contribute to injury-induced neointimal hyperplasia^38,40^. Notably, histone 3 lysine modifications have been shown to participate in VSMC plasticity. For example, H3K4me2 removal in VSMCs results in the loss of differentiation and reduced contractility due to the impaired recruitment of TET2^41^. The overexpression of KDM3a, an H3K9me2-specific demethylase, accelerates neointima formation, whereas KDM3a knockdown reduces neointima formation^33^. In particular, other histone arginine methylations that are primarily mediated by PRMT5 were recently shown to regulate SMC marker gene transcription^39,42^. Together, these studies revealed the pivotal role of histone methylation in the proliferation and migration process of VSMCs and prompted us to investigate whether EHMT2 and its histone methyltransferase activity regulate the VSMC proliferation.

In this study, by constructing an *EHMT2*^SMCKO^ mouse model, we found that *EHMT2* deficiency led to reduced neointima formation. In addition, the downregulation of PCNA and upregulation of SM22α after the genetic deletion or chemical inhibition of EHMT2 indicated that the loss of EHMT2 inhibited the SMC transit into a proliferative state. In addition, it has been reported that EHMT2 is recruited by the transcription factor BACH1 and facilitates the BACH1-mediated modulation of VSMC phenotypic switching^43^. Here, our study further provided explicit and in-depth evidence on how EHMT2 itself participated the VSMC proliferation and neointima formation. Mechanistically, our RNA-seq analysis revealed that, in addition to genes related to the cell cycle and DNA synthesis, the underlying genes are related to cell death and apoptosis, findings that are consistent with the observation that EHMT2 inhibits aortic SMC death by suppressing autophagy activation. Moreover, GO analysis indicated that EHMT2 mediates important biological processes in SMCs, such as cell differentiation and development, the MAPK cascade and the JNK cascade, a finding that is consistent with the well-established role of EHMT2 in cancer cell proliferation^44,45^. Furthermore, the methyltransferase activity required for EHMT2-induced VSMC proliferation might suggest the importance of gene silencing during SMC transdifferentiation and neointima formation.

Furthermore, CUT&Tag screening revealed that GADD45G is di-methylated at H3K9 in its promoter region and may serve as a key mediator of EHMT2’s role in vascular remodeling. GADD45G has been reported to play key roles in the cellular stress response, DNA repair, autoimmunity and cell cycle regulation^46,47^. Specifically, GADD45G acts as a novel tumor suppressor, and GADD45G insufficiency leads to significantly enhanced tumor growth through Janus kinase/signal transducer and activator of transcription 3 activation^48–50^. GADD45G is widely recognized as a tumor suppressor. Loss of GADD45G has been shown to facilitate tumor growth, in part through activation of the JAK–STAT3 pathway. Beyond JAK–STAT3, GADD45G can restrain cell proliferation through multiple signaling cascades^48–50^. For example, in myeloproliferative neoplasms, GADD45G was reported to bind RAC2 to inhibit the RAC2–PAK1–PI3K/AKT axis. In acute myeloid leukemia, GADD45G activates p38 MAPK and thereby downregulates E2F1, leading to impaired DNA repair capacity and reduced proliferation^50^. In addition, GADD45G can interact with MAP3K4 to regulate p38 phosphorylation in astrocytes. However, only a handful of studies have investigated the role of GADD45G in CVD. GADD45G may be involved in myocardial cell death and left ventricular remodeling after myocardial infarction^51^. *GADD45G* overexpression induces significant alterations in cardiac morphology and histology, ultimately leading to cardiac remodeling and heart failure^51^. With respect to the role of GADD45G in the cellular stress response and cell cycle regulation, a regulatory function of GADD45G on the VSMC proliferation and vascular intimal hyperplasia is plausible. Given its established role in stress response and cell cycle regulation, GADD45G is hypothesized to modulate VSMC proliferation and intimal hyperplasia^50^. Its antiproliferative effects have been linked to DNA damage-repair mechanisms, and it can also regulate NF-κB-dependent inflammatory responses in a cell type-specific manner^50^. Collectively, these observations support a broader model in which GADD45G functions as a central integrator of DNA damage repair, proliferative signaling and inflammatory pathways to fine-tune cellular proliferation—potentially including VSMCs during vascular remodeling^50^. This study demonstrates that GADD45G acts as an inhibitor of VSMC proliferation, as indicated by the accelerated neointimal hyperplasia following GADD45G knockdown. Importantly, we provide the first evidence of the epigenetic regulation of GADD45G by histone methyltransferase EHMT2, showing that EHMT2-mediated enrichment of H3K9me2 on GADD45G exons represses its transcriptional activity. Overall, these findings expand the regulatory landscape of GADD45G in cell proliferation by linking its established role in cell cycle control to a previously underappreciated vascular context. These observations further identify the EHMT2–H3K9me2–GADD45G axis as a key epigenetic mechanism driving VSMC proliferation and injury-induced vascular remodeling.

Beyond its well-characterized role in histone methylation, accumulating evidence indicates that EHMT2 also regulates cellular processes through nonhistone mechanisms across diverse biological contexts. EHMT2 has a broad repertoire of nonhistone targets and has been shown to activate the tumor suppressor p53 via a mechanism independent of its methyltransferase activity^52,53^. Similarly, EHMT2 can repress MyoD-dependent transcription by depositing H3K9me2 at MyoD target promoters, and by directly interacting with MyoD and methylating specific lysine residues, thereby constraining its transcriptional activity^54^. EHMT2 has been implicated in promoting de novo DNA methylation^55^. Here, the nonhistone targets of EHMT2 were not explored, and it remains elusive whether EHMT2 directly methylates GADD45G or modulates GADD45G function through nonhistone pathways. Nevertheless, we speculate that nonhistone actions of EHMT2 may synergize with the EHMT2–H3K9me2–GADD45G axis, together forming an integrated regulatory network that shapes VSMC behavior during vascular remodeling.

In conclusion, the aberrant upregulation of EHMT2 expression promotes neointima formation in animal models. EHMT2 acts as a crucial epigenetic regulator of VSMC proliferation and migration in a methyltransferase activity-dependent manner. Mechanistically, EHMT2 controls the histone methylation status at the promoter region of GADD45G and represses GADD45G transcription. Thus, EHMT2 and GADD45G could be promising potential therapeutic targets for the development of novel strategies to treat vascular remodeling.

## Supplementary information

Supplementary Information

## Data Availability

The authors declare that all the data and methods supporting the findings of this study are available in the manuscript or Supplementary Material or from the corresponding authors upon reasonable request.
