# Supplementary material for: EHMT2 aggravates vascular remodeling via epigenetic inhibition of GADD45G
Source: Exp Mol Med. 2026 May 1;58(5):1409–24. doi: 10.1038/s12276-026-01702-6 (PMC13233823; doi:10.1038/s12276-026-01702-6)
Supplement: Supplementary file 1 — Supplementary Information [file 12276_2026_1702_MOESM1_ESM.pdf]

Supplementary Data

EHMT2 aggravates vascular remodeling via epigenetic inhibition of GADD45G

Zelan Wang[1#], Junyong Zhao[1#], Wenjian Luo[1#], Ning Sun[2], Xingyu Ma[1], Fangyuan Zhong[1], Boji Wu[1], Heng Tang[1], Ke Ning[1], Jingyu He[1], Xuhong Wang[1], Kun Zhang[3], Jihang Zhang[1], Chuan Liu[1], Jun Ren[4,5]*, Yan Zhao[6]*, Zhexue Qin[1,7]*

[#] These authors contributed equally to this work.

1 Department of Cardiology, Xinqiao Hospital, Army Medical University, Chongqing 400037, China. 2 Department of Cardiology, University-town Hospital of Chongqing Medical University, Chongqing 401331, China. 3 Department of Pathogenic Biology, College of Basic Medical Sciences, Army Medical University, Chongqing 400038, China. 4 Department of Cardiology, Zhongshan Hospital, Fudan University; Shanghai Institute of Cardiovascular Diseases, Shanghai 200032, China. 5 State Key Laboratory of Cardiovascular Diseases, Zhongshan Hospital, Fudan University, Shanghai 200032 China

6 Department of Microbiology, College of Basic Medical Sciences, Key Laboratory of Microbial Engineering Under the Educational Committee in Chongqing, Army Medical University, Chongqing 400038, China. 7 Basic Research Innovation Center for Acute Radiation Syndrome, Ministry of Education of the People's Republic of China, Chongqing, 400037, China

*** Address for correspondence:**

Zhexue Qin MD, PhD Associate Professor. Email: zhexueqin@126.com, zhexueqin@tmmu.edu.cn

Yan Zhao, PhD Associate Professor. Email: hnyanyanxp@aliyun.com,

amuzhaoyan@tmmu.edu.cn

Jun Ren, PhD Professor. Email: jren_aldh2@outlook.com

Supplemental methods

The authors declare that all data and methods supporting the findings of this study are available in the Context or Supplementary Material or from the corresponding authors on reasonable requests.

1. Antibodies

All the antibodies used in this study are detailed in Supplementary Table 1.

2. Animals

The primers used for mouse genotyping were as follows.

EHMT2 flox-P1: TGCCTATGTGGTCAGCTCAG

EHMT2 flox-P2: GGAGAGATGCAGGACATGGT

Tagln-cre-P3: TCGATGCAACGAGTGATGAG

Tagln-cre-P4: TCCATGAGTGAACGAACCTG

The Sprague-Dawley rats and C57BL/6J mice were obtained from Beijing Huafukang Bioscience (Beijing, China).

3. Quantitative real-time PCR

Total RNA was extracted from vascular smooth muscle cells (VSMCs) or carotid artery tissues using TRIzol reagent (Sangon Biotech, Cat: B511311; Shanghai, China) according to the manufacturer’s instructions. RNA was then reverse transcribed using the PrimeScript RT reagent kit (TaKaRa Biotechnology, Kyoto, Japan). The reverse-transcription products were used for SYBR® Green-based real-time quantitative PCR (RT-qPCR) analysis, conducted on an Applied Biosystems 7500 Real-Time PCR System (Massachusetts, USA). Relative mRNA expression levels were normalized to β-tubulin as the internal housekeeping control. Fold changes were calculated using the comparative Ct method ($2^{-\Delta\Delta Ct}$). Primer sequences are provided in Supplementary Table 2.

4. Western blot assays

Mouse artery tissues and VSMCs were homogenized in an ice-cold RIPA lysis buffer (Thermo Fisher Scientific, Cat: WC321067; Massachusetts, USA) supplemented with SDS-PAGE sample loading buffer (ABclonal, Cat: RM00001; Wuhan, China). Homogenates were centrifuged at 10,000 × g for 20 minutes at 4°C, and the supernatant containing total protein was collected and quantified using a BCA protein assay kit (Beyotime Biotechnology, Cat: P0010; Shanghai,

China). Equal amounts of protein were loaded onto SDS-PAGE gels and were separated by electrophoresis, followed by transfer onto polyvinylidene difluoride (PVDF) membranes (Sigma-Aldrich, Cat: 03010040001; Shanghai, China). Following blocking with 5% nonfat dry milk (Beyotime Biotechnology, Cat: P0216-300g; Shanghai, China), membranes were incubated overnight at 4°C with appropriate primary antibodies. Following washing with Tris-buffered saline containing Tween (Beyotime Biotechnology, Cat: ST-825; Shanghai, China), membranes were probed with horseradish peroxidase-conjugated secondary antibodies (anti-rabbit or anti-mouse, dilution 1:1000; Beyotime Biotechnology, Cat: AS014/AS003; Shanghai, China) for 1 hour at room temperature. Detection was performed using enhanced chemiluminescence substrate (Bioground, Cat: BG0001; Chongqing, China), and protein bands were visualized and quantified using a Bio-Rad imaging system (ImageQuant LAS4000 mini), with β-tubulin serving as the internal control. Results were expressed as relative expression, normalizing to respective β-tubulin expression.

5. Immunofluorescence staining

Frozen sections of mouse carotid artery were fixed in 4% paraformaldehyde (Beyotime Biotechnology, Cat: P0099; Shanghai, China) at room temperature (20°C). Following fixation, sections were permeabilized with an immunofluorescence permeabilization solution (Beyotime Biotechnology, Cat: P0096; Shanghai, China) for 20 minutes and were blocked with an immunofluorescence staining blocking solution (Sangon Biotech, Cat: E674004; Shanghai, China). Primary antibodies were applied to respective sections, prior to incubation at 4°C overnight. After washing with PBS containing 0.1% Tween, sections were incubated at room temperature for 1 hour with secondary antibodies: goat anti-rabbit IgG (H+L) DyLight Alexa 649 (dilution 1:1000; Bioground, Cat: BS10034; Chongqing, China) or goat anti-rabbit IgG (H+L) DyLight Alexa 488 (dilution 1:1000; Bioground, Cat: BS10015; Chongqing, China), diluted in a blocking solution. Nuclei were stained with DAPI (Beyotime Biotechnology, Cat: P0131; Shanghai, China). Images were captured using a Zeiss LSM900 confocal laser scanning microscope (Gottingen, Germany). Integrated optical density (IOD) of positive staining was analyzed using the ImageJ software (NIH, Bethesda, MD, USA) according to the integrated

measurement protocol.

6. Wound-healing assay

VSMCs treated with siRNAs were plated in 6-well plates (BIOFIL, Cat: TCP010006; Guangzhou, China). A wound was introduced to the cell monolayer by gently scraping with a sterile 200 μL micropipette tip. Cells were then imaged immediately following wounding and once again 24 hours later using phase-contrast microscopy. Migration area was quantified by counting the number of pixels in wound regions, using the ImageJ software (NIH, Bethesda, MD, USA).

7. Transwell assay

VSMCs were seeded in the upper chamber of 24-well Transwell plates (BIOFIL, Cat: TCP010024; Guangzhou, China) in medium containing 0.2% FBS. The lower chamber was filled with medium with or without 20 ng/mL platelet-derived growth factor. Cells were allowed to migrate for 12–24 hours. After migration, cells on the lower surface of the membrane were fixed with 4% paraformaldehyde and stained with 0.1% crystal violet solution in 20% methanol (Sangon Biotech, Cat: E607309; Shanghai, China). Six random fields were selected for imaging, and number of migrated cells was quantified using ImageJ software (NIH, Bethesda, MD, USA).

8. VSMC viability and proliferation assay

VSMC proliferation was evaluated using the Cell Counting Kit-8 (CCK-8) assay and Ki-67 immunofluorescence staining. Cell viability and proliferation were assessed using a CCK-8 kit (Beyotime Biotechnology, Cat: C0037; Shanghai, China) according to the manufacturer’s protocol. Absorbance at 450 nm was measured using a microplate reader (ALLSHENG, AMR100; Zhejiang, China). Moreover, Ki-67 expression was evaluated using immunofluorescence using a primary antibody against Ki-67 (dilution 1:2000; Cell Signaling Technology, Cat: 11882s; Massachusetts, USA) and visualized with a fluorescence microscope (Zeiss LSM900; Gottingen, Germany). For EdU incorporation assay, cells were treated with 10 μmol/L EdU for 2 hours. Nuclei were stained with DAPI (Beyotime Biotechnology, Cat: P0131; Shanghai, China). Fold change in Ki-67 expression was calculated by normalizing Ki-67 fluorescence intensity to the fluorescence intensity of respective controls.

9. Propidium Iodide Staining and Flow Cytometry Analysis

Propidium iodide (PI) staining was performed using a flow cytometry kit according to the manufacturer’s instructions. Briefly, single-cell suspensions were harvested and fixed with 70% ethanol. After rehydrating cells in PBS, samples were stained with 0.05 mg/mL propidium iodide and 550 U/mL RNase for 30 minutes at room temperature. Flow cytometry analysis was performed using a Gallios Flow Cytometer (excitation at 488 nm, BeckmanCoulter, CA) to assess the DNA content of the cells.

10. Histological and morphometric analyses

Mice or rat were euthanized using sodium pentobarbital (150 mg/kg) at different time points post-injury. Carotid arteries were harvested following perfusion with circulating fluid, fixed in 4% paraformaldehyde in PBS, and embedded in paraffin for subsequent immunofluorescence and morphometric analyses. Serial cross-sections (8 μm) were cut for both analyses. For morphometric analysis, sections were deparaffinized, rehydrated, and stained with hematoxylin and eosin (Beyotime Biotechnology, Cat: C0105S; Shanghai, China). Neointima formation was quantified by a blinded observer, using the intimal area as the primary measure, with analysis performed using ImageJ software (NIH, Bethesda, MD, USA). The average value was calculated from three independent sections of each arterial sample.

11. Chromatin Immunoprecipitation (ChIP)-qPCR Assay

Cells were fixed with 1% formaldehyde (diluted in PBS) at room temperature for 10 min, and cross-linking was quenched by adding glycine . Cells were then lysed with RIPA buffer and nuclear lysis buffer (both supplemented with 1× protease inhibitor cocktail), and chromatin was sheared to 200-500 bp fragments by sonication. The sheared chromatin was incubated with the indicated antibody or control IgG at 4°C overnight, followed by incubation with protein A/G beads for 2 h. Beads were washed sequentially, and bound complexes were eluted with an elution buffer. Cross-linking was reversed at 65°C, after which samples were treated with RNase A and proteinase K. DNA was purified using a DNA purification kit and analyzed by qPCR using SYBR Green Master Mix and specific primers on a qPCR instrument. Enrichment of target regions was calculated via the $2^{(-\Delta\Delta Ct)}$ method. The ChIP kit (JKR23002A from GeneCreate) was used for these experiments. The primer sequences of the ChIP-qPCR were provided in Supplementary Table 3.

12. Histone methylation assay

A total of 1.5 μg recombinant histones were incubated with 2 μg of GST-tagged G9a in the presence of cold S-adenosyl methionine (SAM, 1 mM) at 30°C for 2 hours. Reactions were conducted in the absence or presence of BIX-01294 or UNC0642 (4 μM).

13. Vector construction and cell transfection

The siRNA or shRNA sequences against rat GADD45G, EHMT2 are listed in Supplementary Table 4.

Supplementary Table 1. Antibody used in this study

Name	Catalog	Supplier	Application	
EHMT2	ab185050	Abcam	WB 1:1000	IF 1:500
H3K9me2	ab176882	Abcam	WB 1:1000	IF 1:500
PCNA	2586S	Cell Signaling	WB 1:2000	IF 1:1000
Ki-67	12075	Cell Signaling	IF 1:500	
SM22α	GT336	Gene Tex	WB 1:1000	IF 1:500
Gadd45g	ER63492	Huabio	WB 1:1000	IF 1:200
Histone	AF0009	Beyotime	WB 1:1000	
CyclinB1	AF1606	Beyotime	WB 1:1000	
CyclinD1	AF1183	Beyotime	WB 1:500	IF 1:200
CDK2	AF1063	Beyotime	WB 1:500	
CDK4	AF2515	Beyotime	WB 1:500	
P21	GB115313	Servicebio	WB 1:500	
β-Tubulin	GB12139	Servicebio	WB 1:1000	
Anti-Rabbit IgG(H+L)	AS014	ABclonal	WB 1:2000	
Anti-Mouse IgG(H+L)	AS003	ABclonal	WB 1:2000	
Anti-Rabbit IgG(H+L) Dylight 649	BS10034	Bioworlde	IF 1:500	
Anti-Mouse IgG(H+L) Dylight 488	BS10015	Bioworlde	IF 1:500	
Anti-Mouse IgG(H+L) Dylight 555	BS21642	Bioworlde	IF 1:500	

Supplementary Table 2. Primers used in this study

Gene	Forward	Reverse
EHMT2	GCAGCTCAATCGCAAGC	TGGGCACATTCTCATAGCC
Gadd45g	CCGCCAAAGTCCTGAATGTG	CGCGCACGATGTCAATGTC
β-actin	AGATCAAGATCATTGCTCCTCCT	ACGCAGCTCAGTAACAGTCC

Supplementary Table 3. Primer sequences used in ChIP-qPCR assays

Gene	Forward	Reverse
Gadd45g	ACTCAGGGACCACGGAGTG	GCTGTAGGGACAATCGAGGC

Supplementary Table 4. The sequences of si-RNAs and sh-RNAs.

Gene	Sense	Anti-sense
**si-*EHMT2***	AGUAACGGGCAUCAAUGC	GCAUUGAUGCCCGUUACU
**si-*Gadd45g***	UGGAUUUGUACCAUUCUUCUG	GAAGAAUGGUACAAAUCCAAG
**sh-*EHMT2***	CcggCACCATGAACATCGACCGAAA CTCGAGTTTCGGTCGATGTTCATG GTGTTTTTg	aattcaaaaaCACCATGAACATCGA CCGAAACTCGAGTTTCGGTCGA TGTTCATGGTG

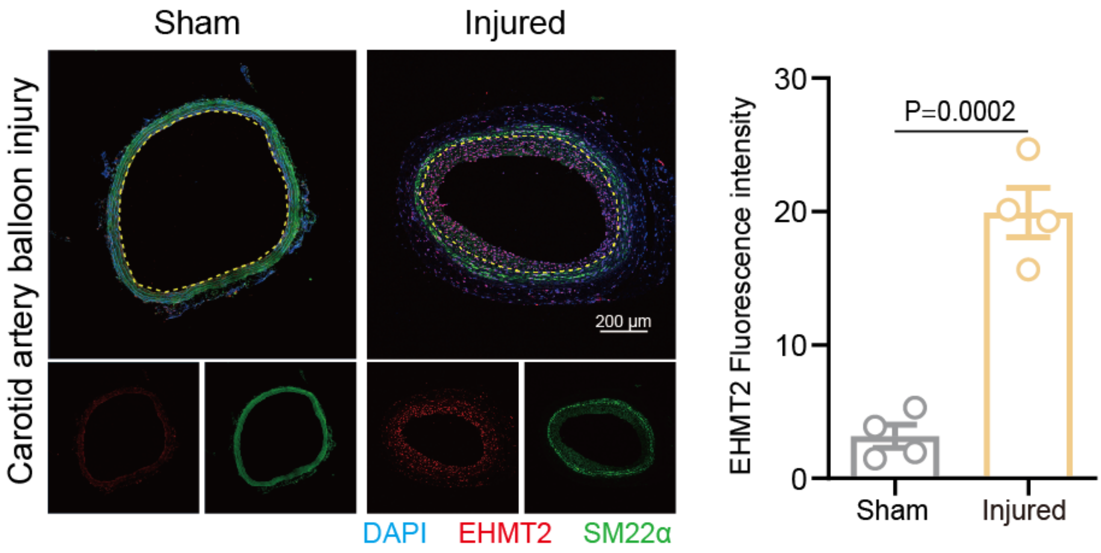

Supplementary Fig. 1 Expression of EHMT2 is elevated in injured arteries. Immunofluorescence staining showed the expression of EHMT2 and SM22α in the carotid arteries (n=4) at day 14 after balloon injury. Nuclei were stained with DAPI. Relative immunofluorescence intensity of EHMT2 was quantified in rat carotid arteries, the intensity was normalized to DAPI. Scale bar = 200μm. P-value were calculated by using an unpaired two-tailed t test. (Extracted the Day 7 staining from Fig. 1a, therefore the sham control figures were shared with Fig. 1a.)

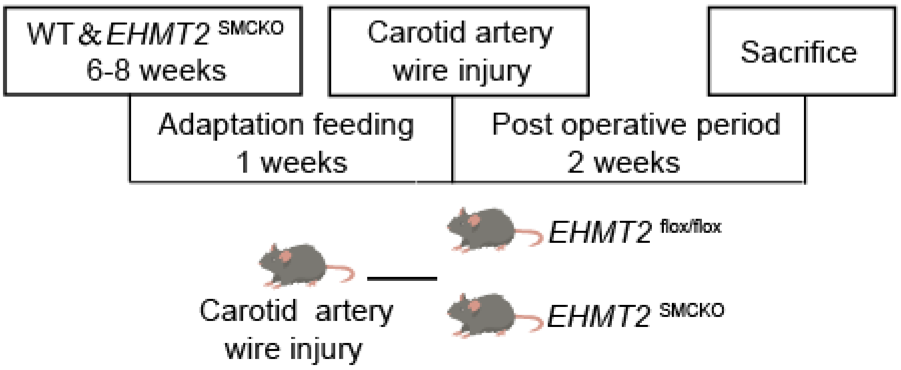

Supplementary Fig. 2 Experimental workflow of carotid artery wire injury in *EHMT2*^{SMCKO} mice. Male mice were subjected to carotid artery wire injury. Postoperatively, mice were assigned to two groups based on their genetic background namely: control group (*EHMT2*$^{flox/flox}$ mice) without specific genetic manipulation, and conditional knockout group (*EHMT2*^{smcko} mice) in which EHMT2 was specifically ablated in smooth muscle cells. At 14 days post-injury, carotid artery neointima tissues were collected from mice in both groups for subsequent analyses.

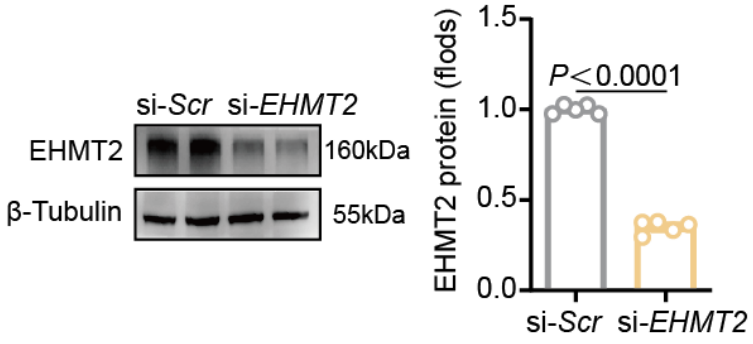

Supplementary Fig. 3 Verification of EHMT2 knockdown efficiency by si-*EHMT2*. The expression of EHMT2 was evaluated via Western blot in rat VSMCs (n=5) treated with si-*EHMT2* or control siRNA. P-value were calculated by using an unpaired two-tailed t test.

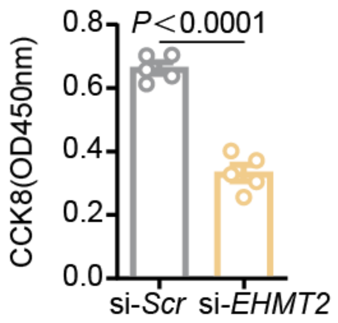

Supplementary Fig. 4 EHMT2 knockdown decreases VSMC viability. VSMCs viability was measured by CCK-8 assay (n=5). VSMCs were stimulated with si-*Scr* or si-*EHMT2*.

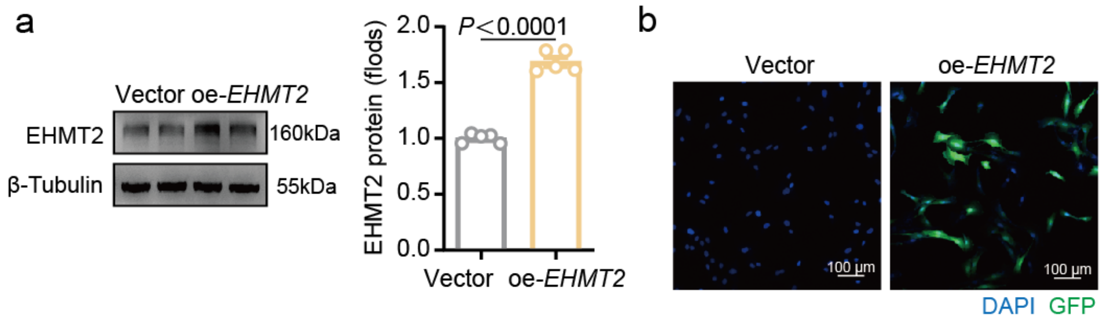

Supplementary Fig. 5 Verification of EHMT2 overexpression efficiency by oe-*EHMT2*. (a) Expression of EHMT2 was evaluated using Western blotting in rat VSMCs (n=5) treated with oe-*EHMT2* or control. (b) The efficiency of transfection with the EHMT2 overexpressing lentivirus was determined using immunofluorescence detection of GFP expression. P-value were calculated using an unpaired two-tailed t test (a).

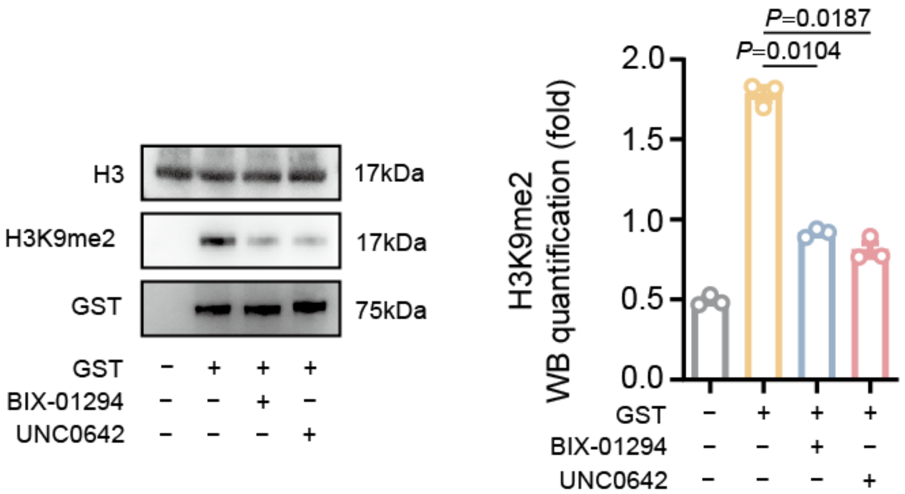

Supplementary Fig. 6 In vitro verification of recombinant EHMT2 enzymatic activity. Enzymatic activity of recombinant GST-EHMT2 toward H3 was measured by western blot analysis using anti-H3K9me2 antibody after incubation of recombinant histone H3 with SAM in the presence or absence of 5 μM inhibitors BIX-01294 or UNC0642 (n=3).

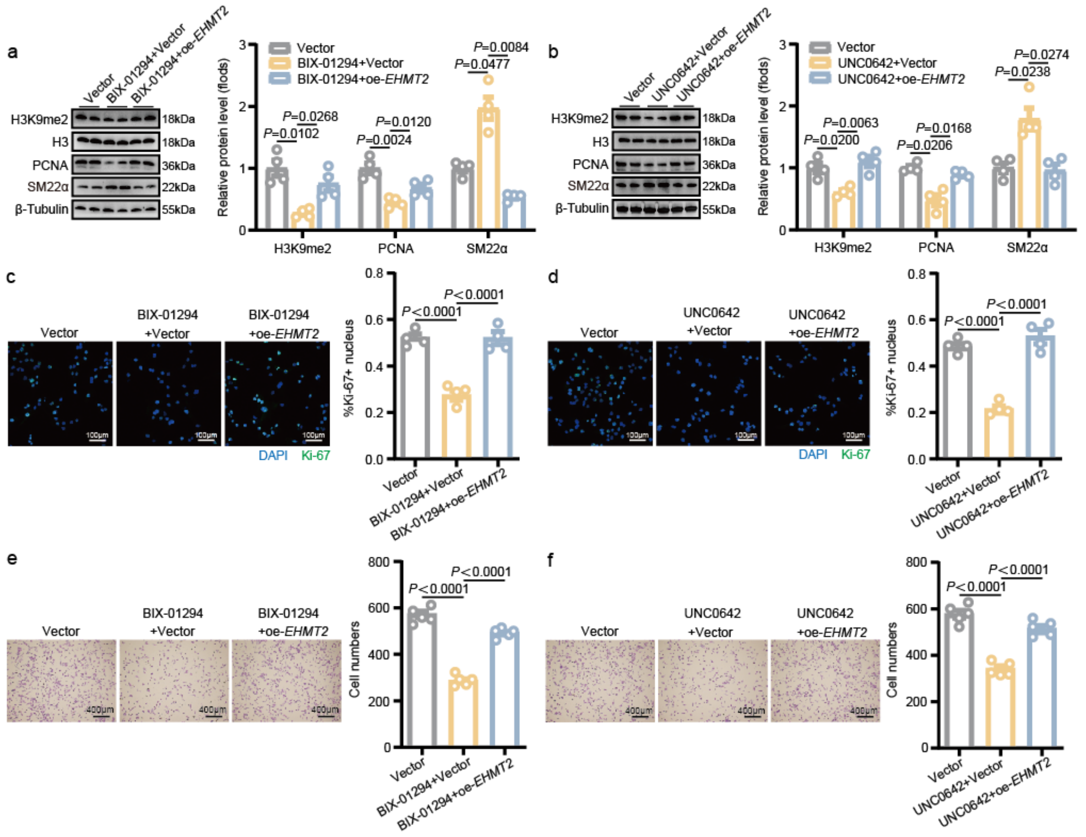

Supplementary Fig. 7 EHMT2-specific inhibitor regulates the proliferation and migration of VSMCs in vitro. (a) Representative Western Blot analysis and quantification of PH3K9me2, PCNA and SM22α in VSMCs treated with vector control, BIX-01294, and BIX-01294 plus oe-*EHMT2* (BIX-01294+oe-*EHMT2*) (n=4 in each group). (b) Representative Western Blot analysis and quantification of PH3K9me2, PCNA and SM22α in VSMCs treated with vector control, UNC0642, and UNC0642 plus oe-*EHMT2* (UNC0642+oe-*EHMT2*) (n=4 in each group). (c) VSMC proliferation was detected via immunostaining of Ki-67 (n=4), and nuclei were stained with DAPI. Scale bar = 100 μm. (d) The proliferation of VSMCs was detected via immunostaining of Ki-67 (n=4), and nuclei were stained with DAPI. Scale bar = 100 μm. (e) Representative images and quantitative analysis of transwell migration assay in the vector control group, BIX-01294 treatment group, and BIX-01294 plus oe-*EHMT2* group (BIX-01294+oe-*EHMT2*) (n=5 in each group). Scale bar = 400 μm. (f) Representative images and quantitative analysis of transwell migration assay in the vector control group, UNC0642 treatment group, and UNC0642 plus oe-*EHMT2* group

(UNC0642+oe-*EHMT2*) (n=5 in each group). Scale bar = 400 μm.

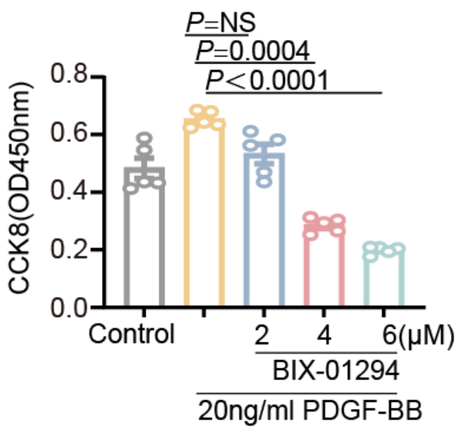

Supplementary Fig. 8 BIX-01294 attenuates PDGF-BB-induced VSMC viability in a dose-dependent manner. VSMCs viability was measured by CCK-8 assay. VSMCs were stimulated with 20 ng/mL PDGF-BB and concurrently treated with varying concentrations (0, 2, 4, 6 μM) BIX-01294. P-value were calculated using a one-way ANOVA.

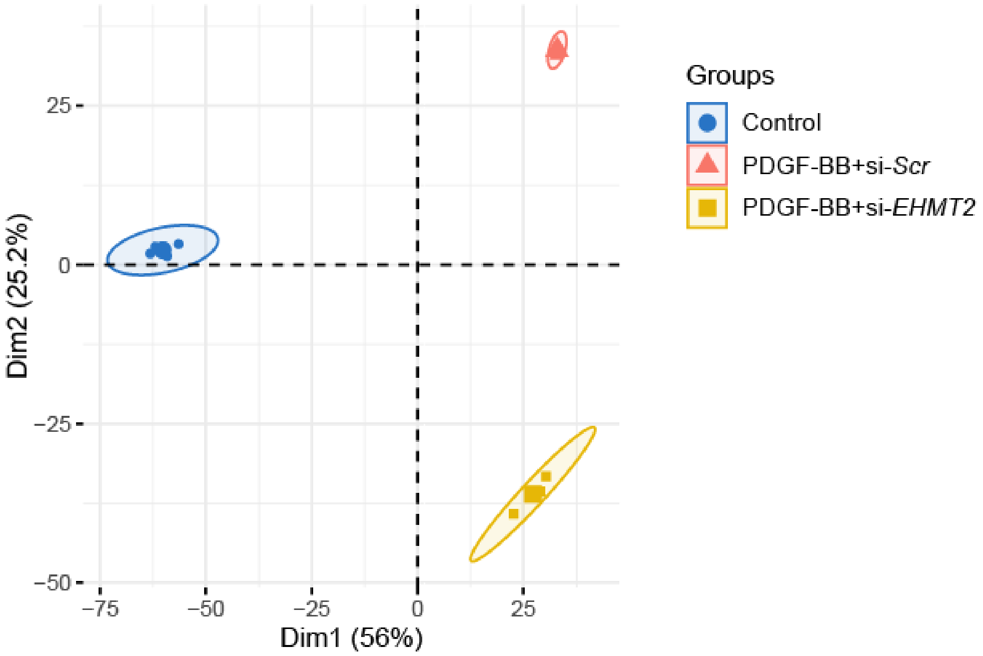

Supplementary Fig. 9 EHMT2 knockdown drives distinct transcriptomic profiles in VSMCs. PCA analysis revealed significant differences in gene expression patterns between VSMCs transfected with the control and EHMT2 knockdown groups in the presence or absence of PDGF-BB.

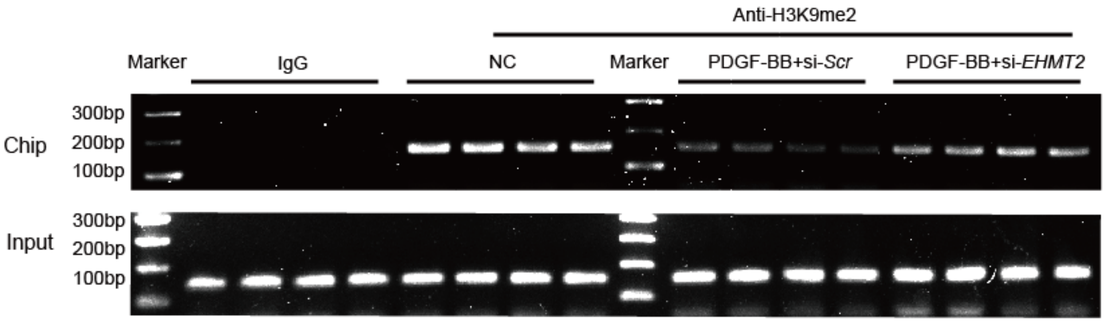

Supplementary Fig. 10 The binding level of H3K9me2 at the promoter region of GADD45G in VSMCs. The PCR products were analyzed on 2% agarose gels and visualized under UV transilluminator. This revealed that H3K9me2 directly binds to the promoter region of Gadd45g in VSMCs.

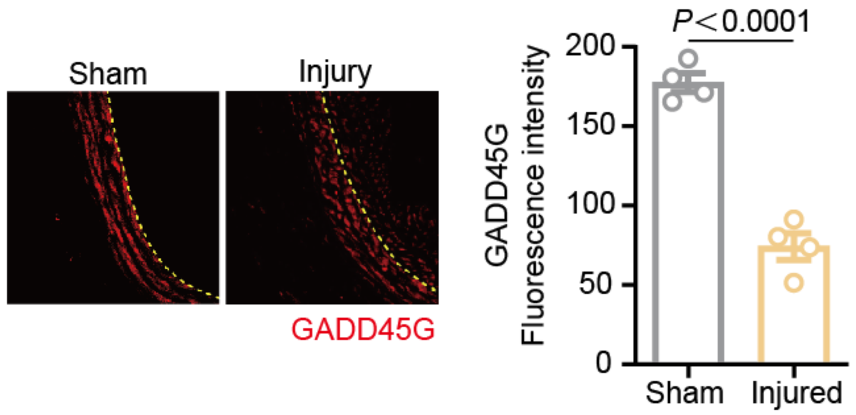

Supplementary Fig. 11 Expression of GADD45G is downregulated in injured arteries. Immunofluorescence staining of GADD45G in the carotid arteries (n=4) at day 14 after balloon injury. Nuclei were stained with DAPI. Scale bar = 200 µm. Relative immunofluorescence intensity of GADD45G was quantified.

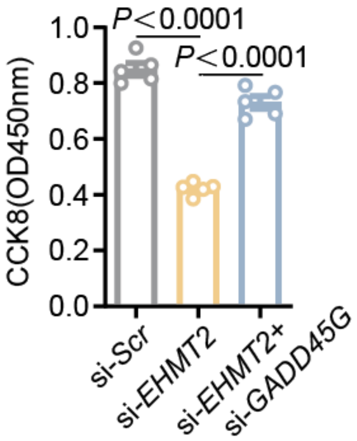

Supplementary Fig. 12 EHMT2 and GADD45G co-regulate VSMC viability. VSMCs viability was measured by CCK-8 assay (n=5). VSMCs were stimulated with with si-*Scr*, si-*EHMT2*, and combined EHMT2/GADD45G knockdown (si-*EHMT2* + si-*GADD45G*).

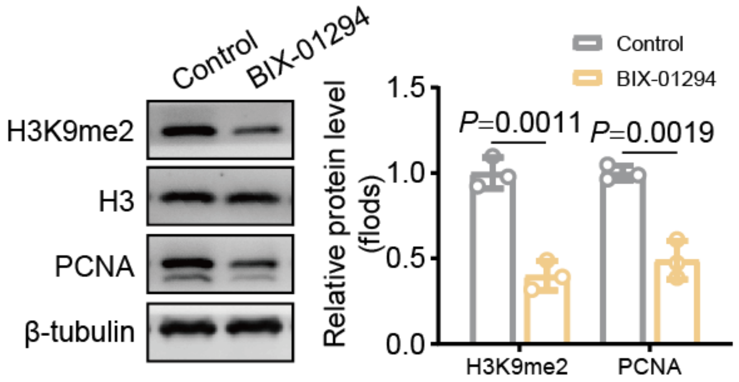

Supplementary Fig. 13 BIX-01294 suppresses H3K9me2 and PCNA expression in vivo. Representative Western blots and quantitative analysis of H3K9me2 and PCNA in the neointima of carotid artery (n=3) at day 14 post-injury in mice treated with or without BIX-01294.
